# Porcine circovirus type 2 (PCV2) evolution before and after the vaccination introduction: A large scale epidemiological study

**DOI:** 10.1038/srep39458

**Published:** 2016-12-19

**Authors:** Giovanni Franzo, Claudia Maria Tucciarone, Mattia Cecchinato, Michele Drigo

**Affiliations:** 1 University of Padua, Legnaro (PD), Italy

## Abstract

Since their commercialization, vaccines against *Porcine circovirus type 2* (PCV2) have been the cornerstone control strategy. Nevertheless, the periodic emergence of new genotype waves and the recent reports of vaccine failure outbreaks have raised the question if widespread vaccination strategies could have driven viral evolution and affected different genotype fitness. To investigate this issue an in-deep analysis, based on a bioinformatics and biostatistics approach, has been implemented. ORF2 sequences from vaccinated and non-vaccinated populations (i.e. domestic pigs before and after vaccine introduction and wild boars) were considered. The action of selective forces on PCV2 strains has been analyzed and compared among groups. Remarkable differences were found in the selective forces acting on viral populations circulating in different “immune environments”. Particularly for PCV2a, a directional selection promoting a change in the viral capsid away from the vaccine specific antigenic determinants has been detected after vaccine introduction. Involved amino acids were previously reported to be part of viral epitopes whose variability is responsible of immune escape. Our findings support a change in PCV2 evolutionary pattern after widespread vaccination introduction and stress once more the compulsoriness of a continuous monitoring of PCV2 epidemiology to promptly act in response to the emergence of possible vaccine-escaping mutants.

Since its discovery in 1991, *Porcine circovirus type 2* (PCV2) has been one of the most present swine virus within the domestic population, reaching seroprevalence levels near to 100%^1^,^2^. Even though PCV2 is a small non-enveloped ssDNA virus with a circular 1766–1768 bp long genome^3^, the complexity of Porcine circovirus diseases (PCVD)^4^ lies in every other trait, from the variety of subclinical and clinical syndromes^5^, to the multifactorial nature of the disease onset^6^, passing through the plethora of transmission routes which help the dissemination and the persistence of the pathogen^7^. Other fascinating aspects of this small virus are the remarkable genetic variability and peculiar evolutionary pathways that have showed to be particularly relevant also in relation to the changes in clinical manifestations occurring through time.

At least five Open Reading Frames (ORF) have been reported to be effectively transcribed, but the most studied and widely sequenced region is the ORF2, which encodes for the Cap protein^3^,^8^,^9^,^10^. This protein represents the only component of the viral capsid and has been proven to be the major target of the host immune response^11^,^12^,^13^. ORF2-based classification criteria^14^,^15^ have been collectively adopted to define PCV2 genotypes because of its higher phylogenetic signal and lower tendency to recombine.

PCV2 geographical distribution and its widespread presence have been undoubtedly linked to livestock movements and trade routes leading to the rapid spread of new strains in various countries^16^.

PCV2 genotype 2a is considered to have been the most prevalent until 2003, when a change in genotype prevalence (commonly known as genotype shift) occurred in favour of genotype 2b^2^, with a parallel enhancement of the outbreak severity^17^,^18^. A similar situation have relapsed since the appearance of the genotype 2d in 2010^19^,^20^, which is rapidly spreading with detriment to PCV2b prevalence^13^. The aforementioned high evolutionary rate (about 10^−3^–10^−4^ substitutions/site/year)^21^ and the huge viral population size provide optimal conditions for natural selection to act. One of the suggested reasons for its wide acquired genetic variability could reside in selective pressures promoted also by vaccination-induced immunity escape^22^.

Since commercial vaccines against PCV2 became commonly available in 2006, their use has increased rapidly thanks to their high efficiency in controlling clinical signs and economical losses^23^. All currently available vaccines, independently from the production techniques (i.e. inactivation, chimeric PCV1-2 virus, Cap subunit expressed in baculovirus system), share the immunogenic capsid protein of the PCV2a genotype^24^. However, in the last decades some concerns have been raised about protection achieved against recently emerged genotypes (i.e. PCV2d). Some published works and many field reports claim episodes of vaccine failure in association with outbreaks of PCV2d^25^,^26^. Further experimental and field trials have disavowed this hypothesis, demonstrating the full protection induced by PCV2a based vaccine against all other genotypes^27^,^28^. Nevertheless, this cannot rule out a certain differential efficacy of current vaccines, which remains irrelevant when animals are vaccinated and raised in optimal conditions, but that can become evident in everyday veterinary practice (i.e. sub-optimal conditions). The role of vaccination in shaping viral evolution has been reported for different diseases affecting both animals and human beings. When immunity is not sterilizing, wild strains are able to circulate in a new “challenging” environment, made of less susceptible-immune hosts. Numerous examples are available of viruses that adapted to this new scenario by immuno-escape (*Hepatitis B virus*^29^, *avian Metapneumovirus*^30^), increase in virulence (Marek’s disease^31^), or both (*Infectious bursal disease virus*^31^). The aforementioned requirements for vaccine evolution and vaccine-induced pathogen replacement are present also for PCV2^32^. Unfortunately, this hypothesis is hard to be demonstrated due to widespread application of vaccines in domestic populations. Kekarainen *et al*.^33^ provided a first evidence of the vaccination role in affecting the genetic variability of PCV2 strains collected from few vaccinating and non-vaccinating farms^33^. However, it is extremely challenging to recruit a significant number of farms where vaccination has not been applied for a long time despite PCV2 circulation. Additionally, contact network between farms is so dense that would be impossible to exclude the recent introduction of strains previously circulating in vaccinated farms^34^.

The present study circumvents these limitation by investigating and comparing selective pressures acting on the capsid protein of PCV2 strains collected from farming animals before and after the vaccine introduction, and on PCV2 strains originating from populations of the same species (*Sus scrofa*) with different vaccination status: the vaccinated domestic pig population and the non-vaccinated wild boar one. Many epidemiological studies have been conducted on PCV2 presence among wild boars, revealing an overall and genotype-specific prevalence comparable to what is commonly found in domestic pigs^35^,^36^,^37^,^38^. Consequently, the wild swine population represents a major source of genetic variability and/or simply of viral exchange, but it is not concerned by the vaccination burden. Therefore the aim of this study was to bring together the available genomic information on wild and domestic pig PCV2 strains to explore their evolutionary pathways in populations with different immune status, to evaluate the potential contribution of genotype 2a-based vaccines in conditioning PCV2 epidemiology and evolution.

## Results

Six databases were obtained by matching and disposing the sequences into six different selected categories, using an imposed time-related cut-off to divide samples collected from non-vaccinated animals (before 2005) and from a period when vaccination was widely adopted (after 2008). Hence we included 112 PCV2a sequences before vaccination (dataset 1), 145 PCV2a sequences after vaccination (dataset 2), 163 PCV2b sequences before vaccination (dataset 3), 84 PCV2b sequences after vaccination (dataset 4), 127 sequences covering the three main PCV2 genotypes (2a, 2b and 2d) from domestic pigs after 2008 (dataset 5), 127 sequences accounting for the same genotypes from wild boars after 2008 (dataset 6). Pairwise genetic distance was evaluated within all datasets and it demonstrated a substantially overlapping pattern between database pairs compared during the study, thus assuring that different dN-dS values were not biased by an unbalanced between-population diversity (Supplementary Figure 1).

**Figure 1:**
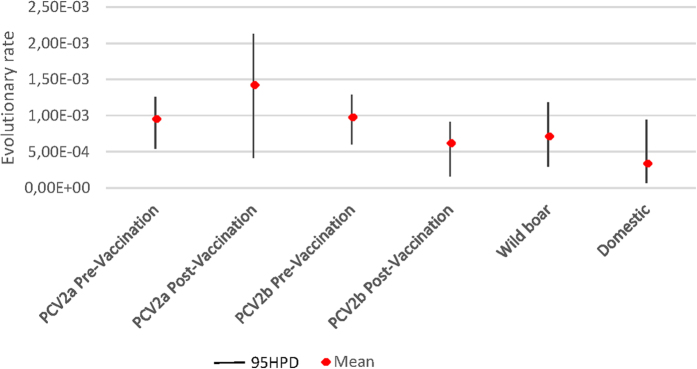
Evolutionary rate (substitution/site/year) calculated for different datasets (i.e. PCV2a before and after vaccination introduction, PCV2b before and after vaccination introduction and PCV2 strains detected in domestic pigs and wild boars). Mean substitution rate and 95HPD interval are reported for each group.

Estimated substitution rate was within the typical range of ssDNA viruses and comparable with what reported by other authors for PCV2^13^,^21^ (Fig. 1). Despite different mean values reported for different dataset, the overlap of 95% Highest Posterior Density (95HPD) supports that the evolutionary rate was the same for all considered populations.

The three main analytic approaches allowed a systematic exploration of selective pressures in action on all the matched databases, with the exception of the domestic and wild population comparison, which did not undergo the directional selection study due to the presence of different “only wild” clades (Supplementary Figure 2). In fact, MEDS was designed with HIV-1 drug resistance in mind and, even if applicable wherever episodic directional selection occurs along multiple lineages, some conditions must be met. One of those is the presence of a rich collection of different background sequences well interspersed in the tree topology. If all the background sequences were so closely related that many foreground and background regions were separated by a single branch, it would be difficult to separate directional selection from founder effects, which would result in a loss of power.

**Figure 2:**
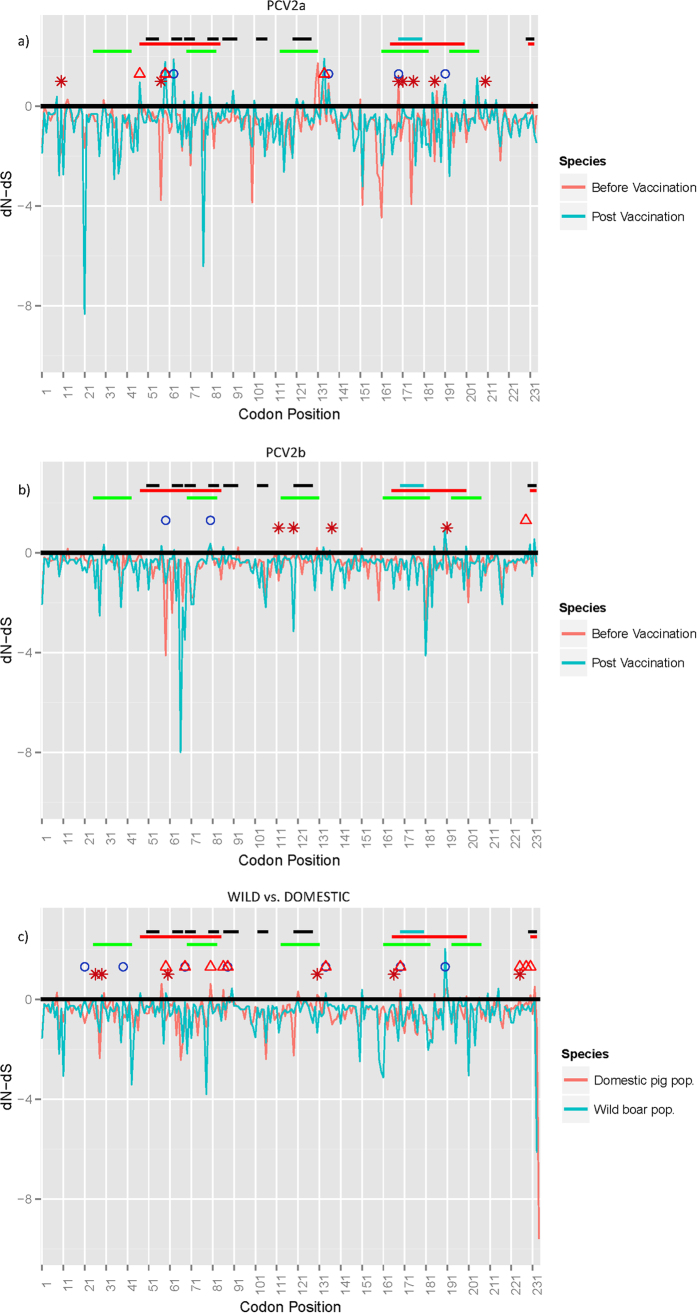
Line graph reporting dN-dS values estimated for each codon position in database 1–2 (i.e. PCV2a before and after vaccination introduction) (**a**), 3–4 (i.e. PCV2b before and after vaccination introduction) (**b**) and 5–6 (PCV2 strains detected in domestic pigs and wild boars) (**c**). For clarity reasons only FUBAR results are reported. Sites detected to be under statistically significant positive selection by MEME or by at least two other methods are reported as colour coded circle (i.e. pre-vaccination groups in a and b and wild boars in c) or triangle (i.e. post-vaccination groups in a and b and domestic pigs in c), while sites detected under different diversifying selection through site-by-site comparison are reported as asterisks. Epitopes experimentally discovered by Trible *et al*.^46^ (blue), Lekcharoensuk *et al*.^47^ (red), Mahe *et al*.^44^ (green), Ge *et al*.^63^ (black) are symbolized by segments.

**Figure 3:**
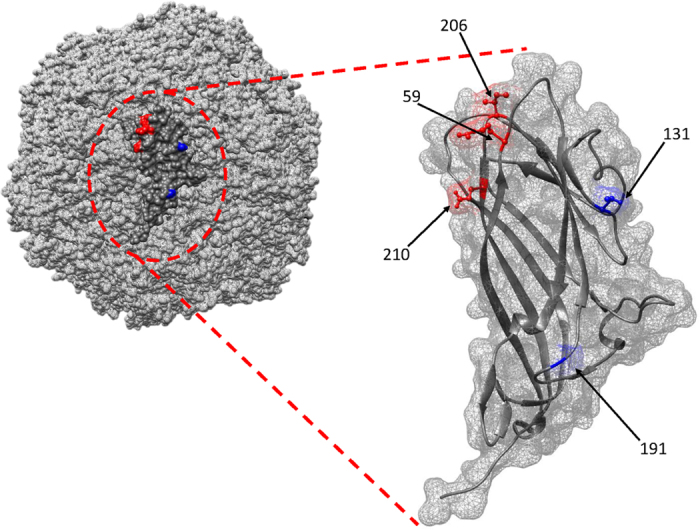
Quaternary and tertiary structure of the PCV2 capsid protein reconstructed by homology modelling. The surface of the capsid and of the single capsomer is represented as a mesh. Amino acid positions detected to be under a directional selection promoting a change in the viral capsid away from the vaccine specific antigenic determinants have been highlighted in red (PCV2a dataset) or blue (PCV2b dataset).

Within the first group (databases 1 and 2), represented by the 2a genotype sequences before and after the vaccination introduction, many sites have been identified as undergoing diversifying selection by either MEME or at least by two other methods among SLAC, FEL and FUBAR (Fig. 2a and Table 1): sites 59, 63, 136, 169 and 191 resulted statistically significant among the before-vaccination PCV2a sequences, while sites 47, 59 and 134 emerged from the after-vaccination PCV2a database. Other sites were proposed as targets of differential selection by site-by-site comparison within the same analyzed data: 10, 57, 169, 171, 176, 186 and 210 (Fig. 2a and Table 2). The study of directional selection by MEDS highlighted the following sites toward the relative amino acids: 59-K, 206-I, 210-E, 232-K (Table 3). The evaluation of the same residues from the Circovac^®^ vaccine strain picked up different amino acids at position 59 (R), 206 (K) and 210 (D), but not at 232 (K) residue (Fig. 3).

In the comparison of databases 3 and 4, two sites (59, 80) from database 3 and one from database 4 (228) appeared under episodic diversifying selection (Fig. 2b and Table 1), whereas four sites (112, 119, 137, 191) showed to be under differential selective pressure (Fig. 2b and Table 2). The investigation of directional pressures revealed two sites, 131 and 191, directed toward P and T respectively (Table 3). Again, strains collected post-vaccination tend to differentiate from Circovac^®^ strain (131-T and 191-D) (Fig. 3).

Wild and domestic populations (database 5 and 6) turned out to share few sites (68, 88, 134, 169) under episodic diversifying selection. Two others (21, 39) were identified only for the wild boar group, while many more (59, 80, 86, 225, 228, 230) were recognised as significant for the domestic pig viral population (Fig. 2c and Table 1). Differential selection appeared to act on sites 26, 29, 60, 130, 166 and 225 (Fig. 2c and Table 2).

## Discussion

Vaccines have been widely used for disease control in both human and animal medicine. Besides the obvious advantages, it is undeniable that vaccination strategies can alter the equilibrium between host and pathogen and modify the competitive hierarchy among viral strains^39^. Vaccine-induced immunity differs from the natural scenario in different ways. At first, it provides a pre-existing immunity that enhances the host resistance to pathogens, potentially increasing the fitness of more virulent strains^40^. Due to the limited number of available vaccine strains, the population immunity is expected to be more homogeneous than in natural conditions, potentially facilitating the emergence of vaccine-escape mutants, not differently from what happens in the case of drug-resistant mutants. This is particularly relevant for high-turnover animal populations, where vaccine-induced immunity plays a pivotal role due to the limited time for natural immune response to develop and act on viral evolution before the subject is removed from the population^31^.

Even if PCV2 is effectively controlled by vaccination, recent episodes of vaccine failure have been reported^25^,^26^,^41^. Although further studies have confirmed the efficacy of current vaccines, at least when applied optimally, some evidences suggesting a differential cross-protection and virulence among strains raised concerns about the threat of PCV2 evolution in response to widespread vaccination implementation^22^,^25^,^26^,^42^. If the vaccination has an effect on viral evolution and/or on the selection of different strains, it is expected that 1) it has an impact on viral population size, 2) it determines an increase in selective pressure, in particular on epitopic regions, 3) it promotes a tendency to evolve distancing from the vaccine sequence itself. While the change over time of different genotypes population dynamics has been reported by several authors^13^,^20^, the other two points have never been examined in detail. A recent study (Reiner *et al*.)^43^ reported the possible effect of vaccination on different genotype prevalence and on PCV2 sequence variation, involving especially an epitopic region located in the viral capsid, by comparing strains obtained from vaccinated and non-vaccinated herds in Germany. Even if an association between some SNP and vaccination status was proven, it is hard to determine if it is actually due to selection or if it is related to the association with particular genotype/clades, to the different genotype prevalence in herds with different status or to the strain temporal evolution. Additionally, the exclusion of correlated SNPs hampered the definition of the genomic positions eventually involved in the escape process^43^. Finally, a widespread between farms PCV2 circulation has been reported in countries with a high pig production^34^ and consequently, even if the precise vaccination status was known in Reiner *et al*.^43^, this paradoxically did not imply that the detected strains had not previously evolved in a vaccinated population.

On the other hand, the present study compares, using a bioinformatics and biostatistics approach, the strength of selective forces acting on viral population collected from different animal populations and time periods, briefly categorizable as “vaccinated” and “non-vaccinated”. Differently from Reiner *et al*.^43^, no epidemiological information were available about the vaccination status of animals included in the study. Nevertheless, it must be stressed that PCV2 vaccines are currently the most sold product in swine farming^24^. Since their introduction, almost if not all pigs are vaccinated directly or indirectly through maternal immunity^24^. Moreover, it has been estimated that 99% of all growing pigs in the USA are vaccinated against PCV2 at/or around the time of weaning^22^. Finally, considering the aforementioned dense PCV2 spreading network among farms and countries, it can be realistically assumed that since the introduction of vaccines, PCV2 strains have been under a vaccine-induced selective pressure.

At first, the effect and strength of evolutionary pressure was evaluated comparing PCV2a field strains collected before and after the introduction of commercial vaccines, also based on PCV2a genotype strains. The evidence that the evolutionary rate was the same for all considered populations suggests that an uneven variability between groups should be attributed to the differential action of selective forces.

Accordingly, site by site comparison reported different sites, located (with the only exception of amino acid 10) in experimentally determined epitopic regions^44^,^45^,^46^,^47^, where selective strength varied between pre and post-vaccination period, being the second one characterized by an increased rate of non-synonymous substitutions. Both populations revealed sites subjected to either pervasive and/or episodic diversifying selection, although the absolute number was lower in the post-vaccination period. Two phenomena can be advocated: sequences belonging to dataset 1 were collected from an expanding and potentially diversifying viral population, while those sampled in the post-vaccination period originated from an already shrinking population, as demonstrated by Shen *et al*.^22^ and Franzo *et al*.^13^,^22^. Another non-conflicting hypothesis suggests that the presence of a population immunity ascribable to an extremely limited number of vaccine strains (and consequently epitopes) could have reduced the tendency to diversification, while prompting a directional change in the viral capsid away from the vaccine specific antigenic determinants. Accordingly to this hypothesis, three out of four amino acids detected under directional episodic selection (all located in epitopes) showed a tendency to mutate toward different amino acids from those of the Circovac^®^ vaccine, the first one to be licensed and the only whose capsid sequence was available. Most interestingly, changes in each of these three amino acids (59-206-210) have been experimentally demonstrated to impair the binding of monoclonal antibodies^44^,^48^. Additionally, in these amino acid positions PCV2a strains tend to mutate toward the amino acid profile of PCV2d (data not showed), for which episodes of vaccine failure have been reported^25^,^26^. Interestingly, when Shen *et al*.^22^ evaluated the prevalence of PCV2a and PCV2b in samples originated from 61 USA sites (both performing or not PCV2 vaccination programs) 5 years after the vaccination introduction, a remarkable difference was observed compared with the pre-vaccination era^22^. Particularly, not only PCV2a was not detected in vaccinated farms but its prevalence in non-vaccinated ones was also lower, with respect to the one estimated before the vaccination introduction. Additionally, by partial sequencing of the capsid gene, some amino-acidic mutations considered potentially favourable for the evasion of the immunity induced by currently used vaccines were observed. It is consequently possible that the introduction of PCV2a- based vaccines dramatically changed the environment where PCV2 circulated, leading to a remarkable shift in the PCV2a population dynamics^13^ and selective forces acting on it. Thus, this and other studies^22^,^43^, provide preliminary but concordant evidences that some of the new PCV2a strains could have the potential to, at least partially, escape from the vaccine strain induced protection. Based on these results, it could be concluded that the three criteria for vaccine-driven selection have so been met for PCV2a.

As expected, PCV2b displayed a less clear, even if comparable, pattern that supports a less marked change in evolutionary dynamics after vaccination introduction. Interestingly, the herein reported mutation in position 131 was also described by Shen *et al*.^22^ in PCV2b strains sampled after vaccination introduction. We decided to evaluate this phenomenon also on the whole PCV2 population by selecting strains of different genotypes collected from different animal populations belonging to the same specie but subjected to different “vaccine pressure” (i.e. wild boars and domestic pigs). The wild boar genotype distribution mirrored the one of the domestic pig, confirming the circulation of each genotype and the rise of PCV2d in the wild population too (Supplementary Figure 2). Also in this case different sites were proven to be under different selective pressure by site-by-site comparison. Nevertheless, just two out of six positions were under a stronger positive selection in domestic pigs, even if it is still suggestive that one of these two (amino acid 130) has been reported to be involved in immuno-escape by Saha *et al*.^48^. Additionally, more sites were estimated to be under diversifying selection in the domestic pig alignment than in the wild one, allowing the speculation of a stronger action of selective pressures in this population. Remarkably, when we evaluated the strength of selective pressures acting on strains collected from 1) wild boars before and after vaccination introduction and 2) wild boars and domestic pigs before vaccination introduction, no significant difference was demonstrated by site-by-site comparison (data not shown). Thus, the genuine effect of vaccine introduction was confirmed to predominate over time and/or host related factors in affecting viral evolution. Unfortunately, the limited amount of data, the unavoidable presence of some methodological assumptions in the used statistical methods, not always perfectly fit by our databases, prevented to conclusively support the role of vaccination in driving non-PCV2a strains evolution.

Nevertheless the results of the present study, combined with the rise of PCV2b and then PCV2d genotypes in a vaccinated population specularly to PCV2a decline, support that the wide application of vaccination could have determined a differential fitness among genotypes, affecting their epidemiology and evolution.

## Conclusion

The present study represents the first in depth analysis and comparison of selective forces acting on PCV2 strains circulating in animal populations with different vaccination status. The results evidence a change between the evolutionary patterns and selective force strength acting on viral populations circulating in vaccinated and non-vaccinated animal populations.

Consequently, it is possible to speculate that, as reported for other pathogens, vaccination strategies could be actually conditioning the evolution and epidemiology of PCV2. The information herein reported provides a strong substratum for further studies and prompts the implementation of complementary researches directed to the understanding of the mechanisms that drive this phenomenon at single animal/local scale level. Additional studies, based on extensive deep sequencing of viral sub-populations sampled over time from animals of known vaccination status, could provide a deeper insight into the basis of PCV2 evolution and confirm the role of vaccination in driving its evolution. Besides providing an interesting model that could be applied to other human and animal diseases, this stresses the compulsoriness of a continuous monitoring of viral epidemiology, particularly for rapidly evolving viruses like PCV2, and the necessity to share the related information to prevent or promptly act in response to the potential emergence of actual vaccine-immunity escape mutants.

## Materials and Methods

### Dataset

A collection of PCV2 ORF2 sequences for which sampling data and host species were available was downloaded from Genbank. The ORF2 gene was selected because it represents the major immunogen of PCV2 and the more variable region of its genome. Additionally, this segment is also the most commonly used for diagnostic and classification purpose and consequently it is the one for which the highest number of sequences, originating from several countries and different host species, is available. The presence of recombination was evaluated using RDP4^49^ and recombinant sequences were excluded from further analysis. Briefly, scan settings were adjusted according to RDP manual and recombination events were accepted if detected by at least 3 method with a p-value >10^−5^ (with Bonferroni correction). Genotypization was then performed using a phylogenetic approach according to Franzo *et al*.^15^ and different databases were created according to genotype, host species and collection data. Considering that vaccination against PCV2 was commonly introduced from 2006 and different vaccines were licensed all over the world by 2008^50^, sequences were divided according to two conservative time points; sequences collected before 2005 were assumed to originate from non-vaccinated animals, while sequences collected after 2008 were considered to originate from vaccinated animals. Although there is no guarantee that all sequences collected after 2008 originated actually from vaccinated animals, the widespread use of vaccination let support the idea that, at least on a regional scale, viruses collected since 2008 have been evolving in a “vaccinated environment”.

PCV2 strains originating from domestic pigs were divided into: 1) PCV2a collected before 2005, 2) PCV2a collected after 2008, 3) PCV2b collected before 2005, 4) PCV2b collected after 2008. Due to the limited number of PCV2d sequences collected before 2005, PCV2d strains were not considered in the present analysis. Similarly, PCV2c was excluded from the study because of its negligible relevance from an epidemiological prospective and the minimum sequence availability. Finally, two other databases (5 and 6 respectively), one for domestic pigs and one for wild boars were set up including sequences of genotypes 2a, 2b and 2d collected after 2008. Unfortunately the limited sequence availability hampered the creation of a fully geographically matched datasets. Even if this was theoretically possible, many sequences collected from specific locations and time periods originated from a limited number of studies investigating substantially homogeneous viral populations. Such artificially unbalanced genetic diversity in different datasets would made difficult to differentiate the phenotypic diversity due to selective pressure action from the one simply caused by genomic variability (i.e. random mutations). For this reasons, efforts were made to stratify sequences in different datasets according to a combination of genotype, geographic origin and category while maintaining a comparable within-dataset genetic distance (Supplementary Tables 1 and 2 and Supplementary Figure 1).

All sequences were aligned at the amino acid level and then the nucleotide sequences were back-translated using the MAFFT algorithm^51^ implemented in TranslatorX^52^.

### Evolutionary rate and analysis of selective pressures

Evolutionary rates were estimated independently for the each dataset using the Bayesian serial-coalescent based method implemented in BEAST 1.8.2^53^. Substitution models were selected based on the Bayesian information criteria (BIC) calculated using Jmodeltest2.1.2^54^ while the best molecular clock was selected based on the Bayesian factor (BF) scores, calculated by estimating the marginal likelihood of different models using both Path Sampling (PS) and Stepping Stone (SS) methods as proposed by Baele *et al*.^55^.

The selective pressure on the viral proteins was evaluated separately for each database (1–6) using different methods based on the estimation of the difference between non-synonymous and synonymous substitution rates (dN-dS). Pervasive and episodic diversifying/purifying selection was estimated using SLAC, FEL, FUBAR and MEME^56^,^57^,^58^. The significance value was set to p < 0.05 for the FEL and MEME methods and to p < 0.1 for SLAC, which claims to be more conservative^59^. The results of FUBAR, were accepted when the posterior probability was greater than 0.9. Sites were assumed to be under diversifying selection when detected by MEME, able to model also episodic diversifying selection, or by at least two other methods. Differences in the site-by-site selection patterns among different databases were investigated for each dataset pair using the batch files *CompareSelectivePressure*.*bf* implemented in HyPhy^60^. To this purposes databases 1–2, 3–4, 5–6 were compared. The presence of episodic directional selection (i.e. when substitutions toward a small number of target amino acids are selected for in at least some part of the tree) was also investigated using MEDS^61^, merging databases 1–2 and 3–4 and setting sequences collected after 2008 as foreground branches. All trees required for the analysis were reconstructed using FastTree^62^.

## Additional Information

**How to cite this article**: Franzo, G. *et al*. Porcine circovirus type 2 (PCV2) evolution before and after the vaccination introduction: A large scale epidemiological study. *Sci. Rep.* **6**, 39458; doi: 10.1038/srep39458 (2016).

**Publisher's note:** Springer Nature remains neutral with regard to jurisdictional claims in published maps and institutional affiliations.

## Supplementary Material

Supplementary Figure 1

Supplementary Figure 2

Supplementary Table 1

Supplementary Table 2

## Figures and Tables

**Table 1:** Table reporting sites detected under statistically significant (in bold) diversifying selection by different methods.

	Codon	SLAC				FEL				MEME				FUBAR			
		*dN-dS* without v.^a^	*p-value* without v.^a^	*dN-dS* with v.^b^	*p-value* with v.^b^	*dN-dS* without v.^a^	*p-value* without v.^a^	*dN-dS* with v.^b^	*p-value* with v.^b^	*ω+* without v.^a^	*p-value* without v.^a^	*ω+* with v.^b^	*p-value* with v.^b^	*dN-dS* without v.^a^	*p-value* without v.^a^	*dN-dS* with v.^b^	*p-value* with v.^b^
PCV2a	47			5.041	**0**.**086**			3.998	**0**.**037**			>100	**0**.**035**			0.555	**0**.**964**
	59	0.595	0.552	2.896	0.339	0.023	0.990	2.995	0.300	46.621	**0**.**000**	37.679	**0**.**000**	0.198	0.622	0.608	0.858
	*63*	5.514	**0**.**062**	3.850	0.281	4.445	**0**.**031**	3.303	0.324	7.091	0.054	2.536	0.322	0.781	**0**.**978**	0.652	**0**.**926**
	72			3.975	0.201			3.304	0.056			>100	0.077			0.435	**0**.**934**
	130	1.211	0.460			2.390	0.262			16.670	0.086			0.528	**0**.**911**		
	133	3.312	0.146			2.052	0.261			5.327	0.216			0.494	**0**.**921**		
	134			2.550	0.385			4.026	0.237			>100	**0**.**000**			0.705	**0**.**924**
	136	3.719	**0**.**051**			2.949	**0**.**009**			>100	**0**.**020**			0.492	**0**.**971**		
	169	0.839	0.515			0.479	0.804			>100	**0**.**000**			0.109	0.634		
	191	4.474	**0**.**057**	4.441	0.166	2.861	**0**.**020**	3.226	0.078	>100	**0**.**011**	>100	0.102	0.521	**0**.**971**	0.383	**0**.**918**
	206	3.685	0.293	4.521	0.281	2.398	0.151	3.745	0.119	>100	0.190	>100	0.148	0.358	**0**.**905**	0.476	**0**.**931**
PCV2b	59	−5.116	0.954			−17.425	0.080			45.823	**0**.**028**						
	80	1.957	0.444			7.287	0.169			>100	**0**.**004**						
	228			1.712	0.760			5.593	0.487			>100	**0**.**036**				
Domestic vs Wild	21	1.020	0.646			0.186	0.949			>100	**0**.**012**						
	39	1.692	0.553			0.269	0.891			>100	**0**.**000**						
	59			0.545	0.388			1.360	0.625			66.010	**0**.**001**				
	68	−0.726	0.757	−1.377	0.983	−0.407	0.816	−3.661	0.055	99.854	**0**.**006**	>100	**0**.**002**				
	80			0.909	0.132			3.213	**0**.**032**			>100	**0**.**033**			0.616	**0**.**903**
	86			0.653	0.318			2.257	0.244			>100	**0**.**042**				
	88	1.560	0.538	0.260	0.533	1.749	0.535	1.385	0.592	>100	**0**.**004**	>100	**0**.**001**				
	134	−1.547	0.772	0.228	0.566	4.650	0.220	1.421	0.336	>100	**0**.**000**	>100	**0**.**015**				
	169	0.496	0.610	0.641	0.292	−1.096	0.772	2.645	0.154	68.979	**0**.**001**	>100	**0**.**000**				
	190													2.018	**0**.**958**		
	225			0.294	0.680			0.835	0.326			>100	**0**.**022**				
	228			0.278	0.759			0.712	0.496			>100	**0**.**010**				
	230			0.272	0.519			1.564	0.251			>100	**0**.**019**				

For each method dN-dS values or ω+ (dN/dS) and the respective p-values are reported.

^a^Without v. = Value obtained for datasets including sequences of PCV2 strains collected from non-vaccinated populations.

^b^With v. = Value obtained for datasets including sequences of PCV2 strains collected from vaccinated populations.

**Table 2:** Table reporting sites where dN/dS difference between the codon positions of the considered alignments was statistically significant (p-value <0.05).

	Codon	Site by site
		*p-value*
PCV2a	10	**0**.**026**
	57	**0**.**051**
	169	**0**.**055**
	171	**0**.**037**
	176	**0**.**055**
	186	**0**.**024**
	210	**0**.**017**
PCV2b	112	**0**.**044**
	119	**0**.**044**
	137	**0**.**054**
	191	**0**.**040**
Domestic vs Wild	26	**0**.**048**
	29	**0**.**053**
	60	**0**.**049**
	130	**0**.**005**
	166	**0**.**042**
	225	**0**.**055**

**Table 3:** Table reporting sites under episodic directional selection, setting sequences collected from vaccinated animals as foreground branches.

	Codon	MEDS	
		AA^a^	*p-value*
PCV2a	59	K	**<0**.**001**
	206	I	**<0**.**001**
	210	E	**<0**.**001**
	232	K	**<0**.**001**
PCV2b	131	P	**<0**.**001**
	228	T	**0**.**001**

^a^Amino acid toward directional selection points.
