# Supplementary material for: Porcine circovirus type 2 (PCV2) evolution before and after the vaccination introduction: A large scale epidemiological study
Source: Sci Rep. 2016 Dec 19;6:39458. doi: 10.1038/srep39458 (PMC5171922; doi:10.1038/srep39458)
Supplement: Supplementary Figure 1 [file srep39458-s1.pdf]

Porcine circovirus type 2 (PCV2) evolution before and after the vaccination introduction. A large scale epidemiological study.

Giovanni Franzo[*#1], Claudia Maria Tucciarone[#1], Mattia Cecchinato[1] and Michele Drigo[1].

[1]University of Padua, Legnaro (PD), Italy;

Supplementary figure 1. Density curves representing pairwise p-distance calculated for dataset 1-2 (a), 3-4 (b) and 5-6 (c).

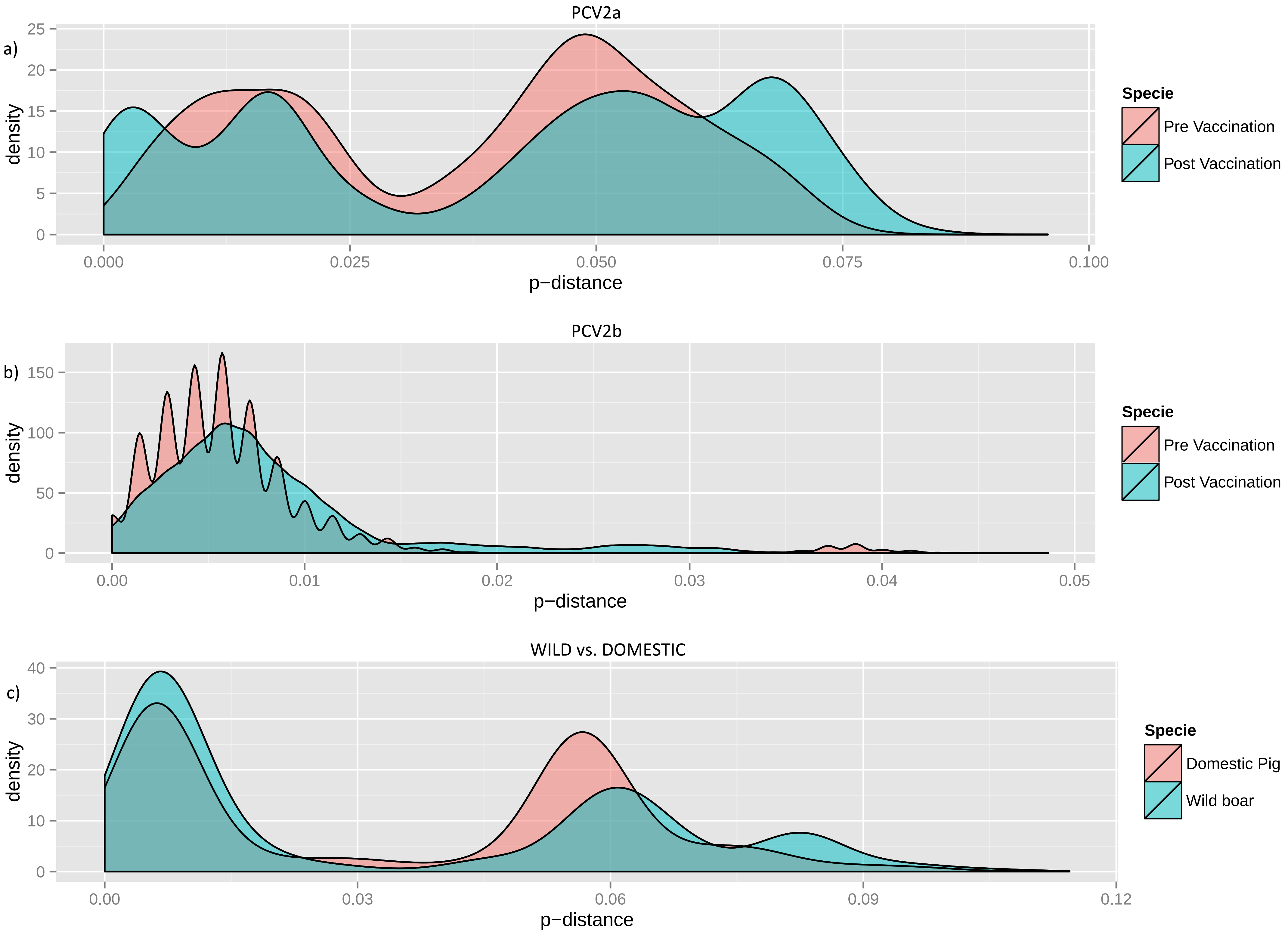

PCV2a
a)
density
p−distance
0.000
0.025
0.050
0.075
0.100
0
5
10
15
20
25
Specie
Pre Vaccination
Post Vaccination
PCV2b
b)
density
p−distance
0.00
0.01
0.02
0.03
0.04
0.05
0
50
100
150
Specie
Pre Vaccination
Post Vaccination
WILD vs. DOMESTIC
c)
density
p−distance
0.00
0.03
0.06
0.09
0.12
0
10
20
30
40
Specie
Domestic Pig
Wild boar
