# Supplementary material for: Porcine circovirus type 2 (PCV2) evolution before and after the vaccination introduction: A large scale epidemiological study
Source: Sci Rep. 2016 Dec 19;6:39458. doi: 10.1038/srep39458 (PMC5171922; doi:10.1038/srep39458)
Supplement: Supplementary Figure 2 [file srep39458-s2.pdf]

Porcine circovirus type 2 (PCV2) evolution before and after the vaccination introduction. A large scale epidemiological study.

Giovanni Franzo[*#1], Claudia Maria Tucciarone[#1], Mattia Cecchinato[1] and Michele Drigo[1].

[1]University of Padua, Legnaro (PD), Italy;

Supplementary figure 2. Maximum likelihood phylogenetic tree reconstructed using a collection of PCV2 ORF2 sequences sampled from wild and domestic pigs. On the left, the whole PCV2 topology is depicted while on the right genotypes specific sub-trees are reported. PCV2 genotypes have been colour-coded in red (PCV2a), blue (PCV2b) and green (PCV2d) while the sequence names of strains collected from wild boars are highlighted in red.

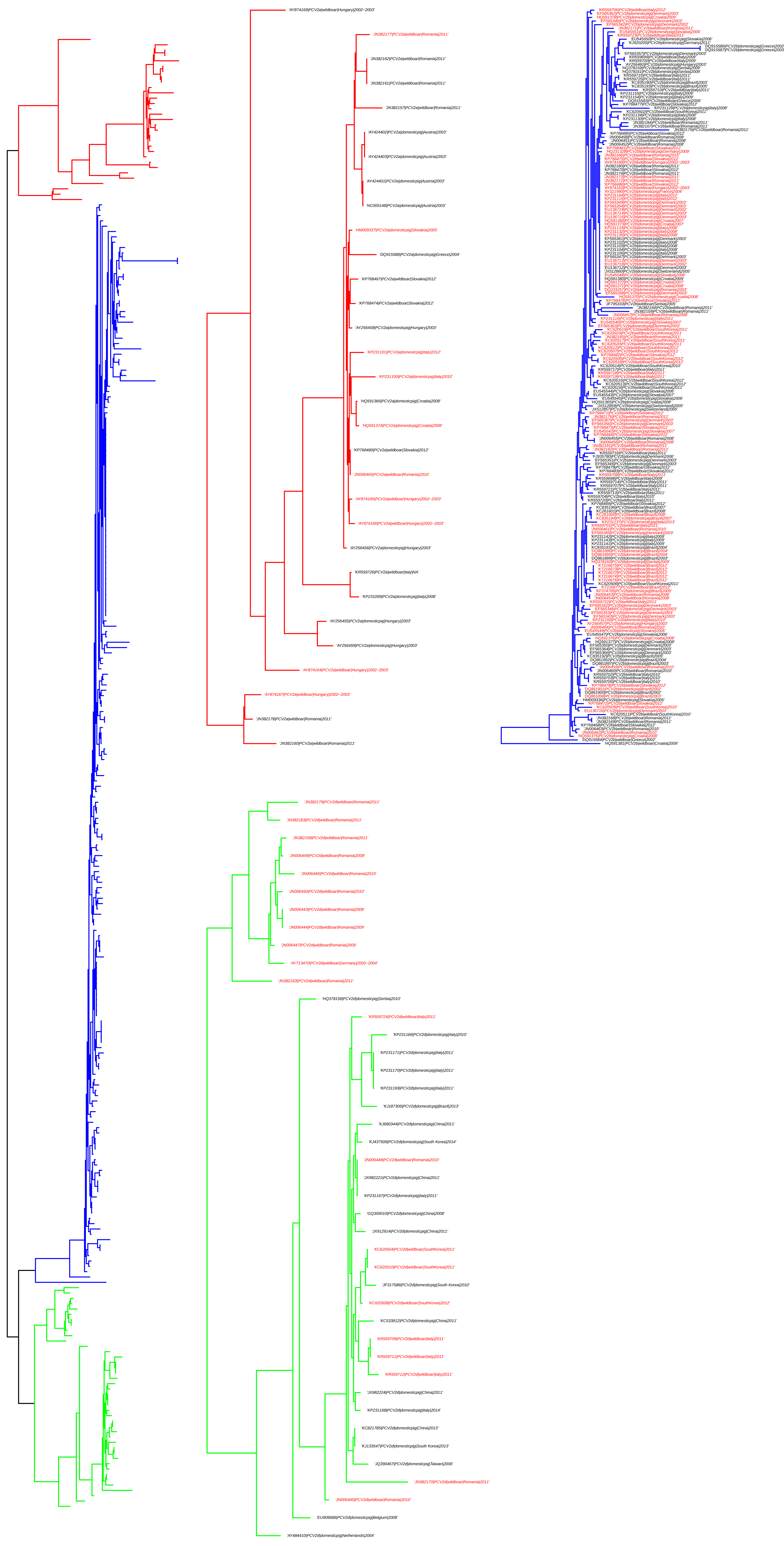
'AY874169|PCV2a|wildboar|Hungary|2002~2003'
'JN382177|PCV2a|wildboar|Romania|2011'
'JN382162|PCV2a|wildboar|Romania|2011'
'JN382161|PCV2a|wildboar|Romania|2011'
'JN382157|PCV2a|wildboar|Romania|2011'
'AY424402|PCV2a|domesticpig|Austria|2003'
'AY424403|PCV2a|domesticpig|Austria|2003'
'AY424401|PCV2a|domesticpig|Austria|2003'
'NC005148|PCV2a|domesticpig|Austria|2003'
'HM009337|PCV2a|domesticpig|Slovakia|2005'
'DQ915588|PCV2a|domesticpig|Greece|2004'
'KP768467|PCV2a|wildboar|Slovakia|2012'
'KP768474|PCV2a|wildboar|Slovakia|2012'
'AY256458|PCV2a|domesticpig|Hungary|2003'
'KP231101|PCV2a|domesticpig|Italy|2012'
'KP231100|PCV2a|domesticpig|Italy|2010'
'HQ591366|PCV2a|domesticpig|Croatia|2008'
'HQ591374|PCV2a|domesticpig|Croatia|2008'
'KP768480|PCV2a|wildboar|Slovakia|2012'
'JN006465|PCV2a|wildboar|Romania|2010'
'AY874165|PCV2a|wildboar|Hungary|2002~2003'
'AY874166|PCV2a|wildboar|Hungary|2002~2003'
'AY256456|PCV2a|domesticpig|Hungary|2003'
'KR559726|PCV2a|wildboar|Italy|NA'
'KP231099|PCV2a|domesticpig|Italy|2008'
'AY256455|PCV2a|domesticpig|Hungary|2003'
'AY256459|PCV2a|domesticpig|Hungary|2003'
'AY874164|PCV2a|wildboar|Hungary|2002~2003'
'AY874167|PCV2a|wildboar|Hungary|2002~2003'
'JN382178|PCV2a|wildboar|Romania|2011'
'JN382160|PCV2a|wildboar|Romania|2011'
'JN382179|PCV2d|wildboar|Romania|2011'
'JN382183|PCV2d|wildboar|Romania|2011'
'JN382158|PCV2d|wildboar|Romania|2011'
'JN006449|PCV2d|wildboar|Romania|2008'
'JN006446|PCV2d|wildboar|Romania|2010'
'JN006450|PCV2d|wildboar|Romania|2010'
'JN006443|PCV2d|wildboar|Romania|2008'
'JN006444|PCV2d|wildboar|Romania|2009'
'JN006447|PCV2d|wildboar|Romania|2009'
'AY713470|PCV2d|wildboar|Germany|2003~2004'
'JN382163|PCV2d|wildboar|Romania|2011'
'HQ378158|PCV2d|domesticpig|Serbia|2010'
'KR559724|PCV2d|wildboar|Italy|2011'
'KP231166|PCV2d|domesticpig|Italy|2010'
'KP231171|PCV2d|domesticpig|Italy|2011'
'KP231170|PCV2d|domesticpig|Italy|2011'
'KP231169|PCV2d|domesticpig|Italy|2011'
'KJ187306|PCV2d|domesticpig|Brazil|2013'
'KJ680344|PCV2d|domesticpig|China|2011'
'KJ437506|PCV2d|domesticpig|South Korea|2014'
'JN006448|PCV2d|wildboar|Romania|2010'
'JX982221|PCV2d|domesticpig|China|2011'
'KP231167|PCV2d|domesticpig|Italy|2011'
'GQ359010|PCV2d|domesticpig|China|2008'
'JX912914|PCV2d|domesticpig|China|2011'
'KC620504|PCV2d|wildboar|SouthKorea|2011'
'KC620510|PCV2d|wildboar|SouthKorea|2011'
'JF317586|PCV2d|domesticpig|South Korea|2010'
'KC620508|PCV2d|wildboar|SouthKorea|2012'
'KC533812|PCV2d|domesticpig|China|2011'
'KR559709|PCV2d|wildboar|Italy|2011'
'KR559711|PCV2d|wildboar|Italy|2011'
'KR559712|PCV2d|wildboar|Italy|2011'
'JX982224|PCV2d|domesticpig|China|2011'
'KP231168|PCV2d|domesticpig|Italy|2014'
'KC821785|PCV2d|domesticpig|China|2013'
'KJ133547|PCV2d|domesticpig|South Korea|2013'
'JQ390467|PCV2d|domesticpig|Taiwan|2006'
'JN382170|PCV2d|wildboar|Romania|2011'
'JN006445|PCV2d|wildboar|Romania|2010'
'EU909686|PCV2d|domesticpig|Belgium|2008'
'AY484410|PCV2d|domesticpig|Netherlands|2004'
