# Supplementary material for: Porcine circovirus type 2 (PCV2) evolution before and after the vaccination introduction: A large scale epidemiological study
Source: Sci Rep. 2016 Dec 19;6:39458. doi: 10.1038/srep39458 (PMC5171922; doi:10.1038/srep39458)
Supplement: Supplementary Table 1 [file srep39458-s3.pdf]

Porcine circovirus type 2 (PCV2) evolution before and after the vaccination introduction. A large scale epidemiological study.

Giovanni Franzo[*#1], Claudia Maria Tucciarone[#1], Mattia Cecchinato[1] and Michele Drigo[1].

[1]University of Padua, Legnaro (PD), Italy;

Supplementary table 1. List of used sequence accession numbers and relative meta-data.

Supplementary table 1

Acc.Number	Coutry	Year	Genotype	Category	Area
EU148506	Denmark	1993	Pcv2a	Pre-Vaccination	Europe
EU386606	Sweden	1993	Pcv2a	Pre-Vaccination	Europe
JX512853	Switzerland	1994	Pcv2a	Pre-Vaccination	Europe
AY754016	Australia	1999	Pcv2a	Pre-Vaccination	Oceania
KC620521	SouthKorea	1999	Pcv2a	Pre-Vaccination	Asia
AF109399	Canada	2000	Pcv2a	Pre-Vaccination	North America
AF117753	Canada	2000	Pcv2a	Pre-Vaccination	North America
AF118095	Canada	2000	Pcv2a	Pre-Vaccination	North America
AF118097	Canada	2000	Pcv2a	Pre-Vaccination	North America
AF201305	Germany	2000	Pcv2a	Pre-Vaccination	Europe
AF201306	Germany	2000	Pcv2a	Pre-Vaccination	Europe
AF201307	Germany	2000	Pcv2a	Pre-Vaccination	Europe
AF201308	Spain	2000	Pcv2a	Pre-Vaccination	Europe
AF201309	Spain	2000	Pcv2a	Pre-Vaccination	Europe
AF201310	Spain	2000	Pcv2a	Pre-Vaccination	Europe
AF264038	USA	2000	Pcv2a	Pre-Vaccination	North America
AF264039	USA	2000	Pcv2a	Pre-Vaccination	North America
AF264040	USA	2000	Pcv2a	Pre-Vaccination	North America
AF264041	USA	2000	Pcv2a	Pre-Vaccination	North America
AF264042	USA	2000	Pcv2a	Pre-Vaccination	North America
AF264043	USA	2000	Pcv2a	Pre-Vaccination	North America
AY754017	Australia	2000	Pcv2a	Pre-Vaccination	Oceania
AY754018	Australia	2000	Pcv2a	Pre-Vaccination	Oceania
AY754019	Australia	2000	Pcv2a	Pre-Vaccination	Oceania
AY754020	Australia	2000	Pcv2a	Pre-Vaccination	Oceania
AY754021	Australia	2000	Pcv2a	Pre-Vaccination	Oceania
JX512854	Switzerland	2000	Pcv2a	Pre-Vaccination	Europe
KC620522	SouthKorea	2000	Pcv2a	Pre-Vaccination	Asia
KC620523	SouthKorea	2000	Pcv2a	Pre-Vaccination	Asia
KC620524	SouthKorea	2000	Pcv2a	Pre-Vaccination	Asia
AB072301	Japan	2001	Pcv2a	Pre-Vaccination	Asia
AB072302	Japan	2001	Pcv2a	Pre-Vaccination	Asia
AB072303	Japan	2001	Pcv2a	Pre-Vaccination	Asia
AF364094	Taiwan	2001	Pcv2a	Pre-Vaccination	Asia
AF381175	China	2001	Pcv2a	Pre-Vaccination	Asia
AF381176	China	2001	Pcv2a	Pre-Vaccination	Asia
AF381177	China	2001	Pcv2a	Pre-Vaccination	Asia
AF408635	Canada	2001	Pcv2a	Pre-Vaccination	North America
KC620525	SouthKorea	2001	Pcv2a	Pre-Vaccination	Asia
KC620526	SouthKorea	2001	Pcv2a	Pre-Vaccination	Asia
KC620527	SouthKorea	2001	Pcv2a	Pre-Vaccination	Asia
KC620528	SouthKorea	2001	Pcv2a	Pre-Vaccination	Asia
AF454546	SouthKorea	2002	Pcv2a	Pre-Vaccination	Asia
AF520783	SouthKorea	2002	Pcv2a	Pre-Vaccination	Asia
AF544024	Korea	2002	Pcv2a	Pre-Vaccination	Asia
AY146991	Taiwan	2002	Pcv2a	Pre-Vaccination	Asia
AY146993	Taiwan	2002	Pcv2a	Pre-Vaccination	Asia
AY754022	Australia	2002	Pcv2a	Pre-Vaccination	Oceania
AY874164	Hungary	2002	Pcv2a	Pre-Vaccination	Europe
AY874165	Hungary	2002	Pcv2a	Pre-Vaccination	Europe
AF465211	Taiwan	2002	Pcv2a	Pre-Vaccination	Asia
KC620529	SouthKorea	2002	Pcv2a	Pre-Vaccination	Asia

KT868337	USA	2002	Pcv2a	Pre-Vaccination	North America
AY094619	USA	2003	Pcv2a	Pre-Vaccination	North America
AY180396	Taiwan	2003	Pcv2a	Pre-Vaccination	Asia
AY180397	Taiwan	2003	Pcv2a	Pre-Vaccination	Asia
AY181948	China	2003	Pcv2a	Pre-Vaccination	Asia
AY256455	Hungary	2003	Pcv2a	Pre-Vaccination	Europe
AY256456	Hungary	2003	Pcv2a	Pre-Vaccination	Europe
AY256458	Hungary	2003	Pcv2a	Pre-Vaccination	Europe
AY256459	Hungary	2003	Pcv2a	Pre-Vaccination	Europe
AY288135	China	2003	Pcv2a	Pre-Vaccination	Asia
AY325495	SouthAfrica	2003	Pcv2a	Pre-Vaccination	Africa
AY424401	Austria	2003	Pcv2a	Pre-Vaccination	Europe
AY424402	Austria	2003	Pcv2a	Pre-Vaccination	Europe
AY424403	Austria	2003	Pcv2a	Pre-Vaccination	Europe
AY874166	Hungary	2003	Pcv2a	Pre-Vaccination	Europe
AY874169	Hungary	2003	Pcv2a	Pre-Vaccination	Europe
DQ397521	USA	2003	Pcv2a	Pre-Vaccination	North America
EU136711	Denmark	2003	Pcv2a	Pre-Vaccination	Europe
KC620530	SouthKorea	2003	Pcv2a	Pre-Vaccination	Asia
KT868318	USA	2003	Pcv2a	Pre-Vaccination	North America
KT868430	USA	2003	Pcv2a	Pre-Vaccination	North America
NC005148	Austria	2003	Pcv2a	Pre-Vaccination	Europe
AY322004	France	2004	Pcv2a	Pre-Vaccination	Europe
AY556474	China	2004	Pcv2a	Pre-Vaccination	Asia
AY699793	USA	2004	Pcv2a	Pre-Vaccination	North America
DQ915588	Greece	2004	Pcv2a	Pre-Vaccination	Europe
KC620531	SouthKorea	2004	Pcv2a	Pre-Vaccination	Asia
KC620532	SouthKorea	2004	Pcv2a	Pre-Vaccination	Asia
KT868051	USA	2004	Pcv2a	Pre-Vaccination	North America
KT868310	USA	2004	Pcv2a	Pre-Vaccination	North America
KT868311	USA	2004	Pcv2a	Pre-Vaccination	North America
KT868314	USA	2004	Pcv2a	Pre-Vaccination	North America
DQ104421	China	2005	Pcv2a	Pre-Vaccination	Asia
DQ104423	China	2005	Pcv2a	Pre-Vaccination	Asia
DQ629113	USA	2005	Pcv2a	Pre-Vaccination	North America
DQ629114	USA	2005	Pcv2a	Pre-Vaccination	North America
DQ870484	USA	2005	Pcv2a	Pre-Vaccination	North America
EF394774	Canada	2005	Pcv2a	Pre-Vaccination	North America
EF394775	Canada	2005	Pcv2a	Pre-Vaccination	North America
EF394776	Canada	2005	Pcv2a	Pre-Vaccination	North America
FJ483938	China	2005	Pcv2a	Pre-Vaccination	Asia
HM009337	Slovakia	2005	Pcv2a	Pre-Vaccination	Europe
KT868027	USA	2005	Pcv2a	Pre-Vaccination	North America
KT868033	USA	2005	Pcv2a	Pre-Vaccination	North America
KT868044	USA	2005	Pcv2a	Pre-Vaccination	North America
KT868058	USA	2005	Pcv2a	Pre-Vaccination	North America
KT868060	USA	2005	Pcv2a	Pre-Vaccination	North America
KT868064	USA	2005	Pcv2a	Pre-Vaccination	North America
KT868070	USA	2005	Pcv2a	Pre-Vaccination	North America
KT868315	USA	2005	Pcv2a	Pre-Vaccination	North America
KT868332	USA	2005	Pcv2a	Pre-Vaccination	North America
KT868333	USA	2005	Pcv2a	Pre-Vaccination	North America
KT868334	USA	2005	Pcv2a	Pre-Vaccination	North America

KT868335	USA	2005	Pcv2a	Pre-Vaccination	North America
KT868362	USA	2005	Pcv2a	Pre-Vaccination	North America
KT868366	USA	2005	Pcv2a	Pre-Vaccination	North America
KT868370	USA	2005	Pcv2a	Pre-Vaccination	North America
KT868374	USA	2005	Pcv2a	Pre-Vaccination	North America
KT868378	USA	2005	Pcv2a	Pre-Vaccination	North America
KT868382	USA	2005	Pcv2a	Pre-Vaccination	North America
KT868382	USA	2005	Pcv2a	Pre-Vaccination	North America
KP231099	Italy	2008	Pcv2a	Post-Vaccination	Europe
FJ870967	China	2008	Pcv2a	Post-Vaccination	Asia
FJ870968	China	2008	Pcv2a	Post-Vaccination	Asia
GQ359003	China	2008	Pcv2a	Post-Vaccination	Asia
HM038034	China	2008	Pcv2a	Post-Vaccination	Asia
HQ402903	China	2008	Pcv2a	Post-Vaccination	Asia
HQ591366	Croatia	2008	Pcv2a	Post-Vaccination	Europe
HQ591374	Croatia	2008	Pcv2a	Post-Vaccination	Europe
HQ831519	Portugal	2008	Pcv2a	Post-Vaccination	Europe
HQ831520	Portugal	2008	Pcv2a	Post-Vaccination	Europe
HQ831522	Portugal	2008	Pcv2a	Post-Vaccination	Europe
HQ831536	Portugal	2008	Pcv2a	Post-Vaccination	Europe
KC618389	USA	2008	Pcv2a	Post-Vaccination	North America
KT868452	USA	2008	Pcv2a	Post-Vaccination	North America
KT868479	USA	2008	Pcv2a	Post-Vaccination	North America
KT868482	USA	2008	Pcv2a	Post-Vaccination	North America
KT868491	USA	2008	Pcv2a	Post-Vaccination	North America
GU001709	China	2009	Pcv2a	Post-Vaccination	Asia
GU370063	China	2009	Pcv2a	Post-Vaccination	Asia
GU370064	China	2009	Pcv2a	Post-Vaccination	Asia
HQ378164	Serbia	2009	Pcv2a	Post-Vaccination	Europe
HQ378165	Serbia	2009	Pcv2a	Post-Vaccination	Europe
HQ831539	Portugal	2009	Pcv2a	Post-Vaccination	Europe
JN133305	Slovakia	2009	Pcv2a	Post-Vaccination	Europe
KF850458	China	2009	Pcv2a	Post-Vaccination	Asia
KT868285	USA	2009	Pcv2a	Post-Vaccination	North America
KT868418	USA	2009	Pcv2a	Post-Vaccination	North America
KP231100	Italy	2010	Pcv2a	Post-Vaccination	Europe
HQ378166	Serbia	2010	Pcv2a	Post-Vaccination	Europe
JF317584	SouthKorea	2010	Pcv2a	Post-Vaccination	Asia
JF682791	China	2010	Pcv2a	Post-Vaccination	Asia
KC800634	China	2010	Pcv2a	Post-Vaccination	Asia
KJ094600	Brazil	2010	Pcv2a	Post-Vaccination	South America
KJ094601	Brazil	2010	Pcv2a	Post-Vaccination	South America
KJ094603	Brazil	2010	Pcv2a	Post-Vaccination	South America
JF682793	China	2011	Pcv2a	Post-Vaccination	Asia
JF682794	China	2011	Pcv2a	Post-Vaccination	Asia
JF928003	China	2011	Pcv2a	Post-Vaccination	Asia
JN639856	China	2011	Pcv2a	Post-Vaccination	Asia
JX948774	China	2011	Pcv2a	Post-Vaccination	Asia
JX982223	China	2011	Pcv2a	Post-Vaccination	Asia
JX982225	China	2011	Pcv2a	Post-Vaccination	Asia
KC800635	China	2011	Pcv2a	Post-Vaccination	Asia
KF850466	China	2011	Pcv2a	Post-Vaccination	Asia
KF850468	China	2011	Pcv2a	Post-Vaccination	Asia

KM191038	USA	2011	Pcv2a	Post-Vaccination	North America
KM191039	USA	2011	Pcv2a	Post-Vaccination	North America
KM191040	USA	2011	Pcv2a	Post-Vaccination	North America
KM191041	USA	2011	Pcv2a	Post-Vaccination	North America
KM191127	Mexico	2011	Pcv2a	Post-Vaccination	South America
KM191128	Mexico	2011	Pcv2a	Post-Vaccination	South America
KT868029	USA	2011	Pcv2a	Post-Vaccination	North America
KT868034	USA	2011	Pcv2a	Post-Vaccination	North America
KP231101	Italy	2012	Pcv2a	Post-Vaccination	Europe
KC188796	SouthKorea	2012	Pcv2a	Post-Vaccination	Asia
KC514989	China	2012	Pcv2a	Post-Vaccination	Asia
KC514996	China	2012	Pcv2a	Post-Vaccination	Asia
KC515002	China	2012	Pcv2a	Post-Vaccination	Asia
KC515024	China	2012	Pcv2a	Post-Vaccination	Asia
KC515025	China	2012	Pcv2a	Post-Vaccination	Asia
KC800636	China	2012	Pcv2a	Post-Vaccination	Asia
KF027495	China	2012	Pcv2a	Post-Vaccination	Asia
KJ680365	China	2012	Pcv2a	Post-Vaccination	Asia
KJ729073	India	2012	Pcv2a	Post-Vaccination	Asia
KM190998	USA	2012	Pcv2a	Post-Vaccination	North America
KT867992	USA	2012	Pcv2a	Post-Vaccination	North America
KT867995	USA	2012	Pcv2a	Post-Vaccination	North America
KT867997	USA	2012	Pcv2a	Post-Vaccination	North America
KT867998	USA	2012	Pcv2a	Post-Vaccination	North America
KT867999	USA	2012	Pcv2a	Post-Vaccination	North America
KT868000	USA	2012	Pcv2a	Post-Vaccination	North America
KT868001	USA	2012	Pcv2a	Post-Vaccination	North America
KT868049	USA	2012	Pcv2a	Post-Vaccination	North America
KT868373	USA	2012	Pcv2a	Post-Vaccination	North America
KT868377	USA	2012	Pcv2a	Post-Vaccination	North America
KT868381	USA	2012	Pcv2a	Post-Vaccination	North America
KT868384	USA	2012	Pcv2a	Post-Vaccination	North America
KT868385	USA	2012	Pcv2a	Post-Vaccination	North America
KT868388	USA	2012	Pcv2a	Post-Vaccination	North America
KT868390	USA	2012	Pcv2a	Post-Vaccination	North America
KT868392	USA	2012	Pcv2a	Post-Vaccination	North America
KT868393	USA	2012	Pcv2a	Post-Vaccination	North America
KT868394	USA	2012	Pcv2a	Post-Vaccination	North America
KT868395	USA	2012	Pcv2a	Post-Vaccination	North America
KT868396	USA	2012	Pcv2a	Post-Vaccination	North America
KT868397	USA	2012	Pcv2a	Post-Vaccination	North America
KT868398	USA	2012	Pcv2a	Post-Vaccination	North America
KT868399	USA	2012	Pcv2a	Post-Vaccination	North America
KT868400	USA	2012	Pcv2a	Post-Vaccination	North America
KT868401	USA	2012	Pcv2a	Post-Vaccination	North America
KT868402	USA	2012	Pcv2a	Post-Vaccination	North America
KT868403	USA	2012	Pcv2a	Post-Vaccination	North America
KT868404	USA	2012	Pcv2a	Post-Vaccination	North America
KT868405	USA	2012	Pcv2a	Post-Vaccination	North America
KT868406	USA	2012	Pcv2a	Post-Vaccination	North America
KT868407	USA	2012	Pcv2a	Post-Vaccination	North America
KT868408	USA	2012	Pcv2a	Post-Vaccination	North America
KT868409	USA	2012	Pcv2a	Post-Vaccination	North America

KT868410	USA	2012	Pcv2a	Post-Vaccination	North America
KT868411	USA	2012	Pcv2a	Post-Vaccination	North America
KT868412	USA	2012	Pcv2a	Post-Vaccination	North America
KT868413	USA	2012	Pcv2a	Post-Vaccination	North America
KT868435	USA	2012	Pcv2a	Post-Vaccination	North America
KT868440	USA	2012	Pcv2a	Post-Vaccination	North America
KM191054	USA	2013	Pcv2a	Post-Vaccination	North America
KM191069	USA	2013	Pcv2a	Post-Vaccination	North America
KM191109	USA	2013	Pcv2a	Post-Vaccination	North America
KM191118	USA	2013	Pcv2a	Post-Vaccination	North America
KM191122	USA	2013	Pcv2a	Post-Vaccination	North America
KM191123	USA	2013	Pcv2a	Post-Vaccination	North America
KM360057	China	2013	Pcv2a	Post-Vaccination	Asia
KP081543	China	2013	Pcv2a	Post-Vaccination	Asia
KP245929	China	2013	Pcv2a	Post-Vaccination	Asia
KP670420	China	2013	Pcv2a	Post-Vaccination	Asia
KT867860	USA	2013	Pcv2a	Post-Vaccination	North America
KT867862	USA	2013	Pcv2a	Post-Vaccination	North America
KT867869	USA	2013	Pcv2a	Post-Vaccination	North America
KT867903	USA	2013	Pcv2a	Post-Vaccination	North America
KT867923	USA	2013	Pcv2a	Post-Vaccination	North America
KT867924	USA	2013	Pcv2a	Post-Vaccination	North America
KT867925	USA	2013	Pcv2a	Post-Vaccination	North America
KT867926	USA	2013	Pcv2a	Post-Vaccination	North America
KT867927	USA	2013	Pcv2a	Post-Vaccination	North America
KT867928	USA	2013	Pcv2a	Post-Vaccination	North America
KT867929	USA	2013	Pcv2a	Post-Vaccination	North America
KT867930	USA	2013	Pcv2a	Post-Vaccination	North America
KT867931	USA	2013	Pcv2a	Post-Vaccination	North America
KT867932	USA	2013	Pcv2a	Post-Vaccination	North America
KT867933	USA	2013	Pcv2a	Post-Vaccination	North America
KT867934	USA	2013	Pcv2a	Post-Vaccination	North America
KT867952	USA	2013	Pcv2a	Post-Vaccination	North America
KT868369	USA	2013	Pcv2a	Post-Vaccination	North America
KT868436	USA	2013	Pcv2a	Post-Vaccination	North America
KM035762	China	2014	Pcv2a	Post-Vaccination	Asia
KM067385	China	2014	Pcv2a	Post-Vaccination	Asia
KM880087	China	2014	Pcv2a	Post-Vaccination	Asia
KM880088	China	2014	Pcv2a	Post-Vaccination	Asia
KM880089	China	2014	Pcv2a	Post-Vaccination	Asia
KP081549	China	2014	Pcv2a	Post-Vaccination	Asia
KP081551	China	2014	Pcv2a	Post-Vaccination	Asia
KR058357	China	2014	Pcv2a	Post-Vaccination	Asia
KT284886	China	2015	Pcv2a	Post-Vaccination	Asia
KT284887	China	2015	Pcv2a	Post-Vaccination	Asia
KT284888	China	2015	Pcv2a	Post-Vaccination	Asia
KT284889	China	2015	Pcv2a	Post-Vaccination	Asia
AF201311	France	2000	Pcv2b	Pre-Vaccination	Europe
AF201897	Netherlands	2000	Pcv2b	Pre-Vaccination	Europe
DQ861900	Brazil	2002	Pcv2b	Pre-Vaccination	South America
DQ861901	Brazil	2002	Pcv2b	Pre-Vaccination	South America
DQ915586	Greece	2002	Pcv2b	Pre-Vaccination	Europe
FJ644919	China	2002	Pcv2b	Pre-Vaccination	Asia

EU136720	Denmark	2002	Pcv2b	Pre-Vaccination	Europe
EU136718	Denmark	2002	Pcv2b	Pre-Vaccination	Europe
EU136719	Denmark	2002	Pcv2b	Pre-Vaccination	Europe
EF565362	Denmark	2003	Pcv2b	Pre-Vaccination	Europe
AY424404	Austria	2003	Pcv2b	Pre-Vaccination	Europe
EF565366	Denmark	2003	Pcv2b	Pre-Vaccination	Europe
EF565355	Denmark	2003	Pcv2b	Pre-Vaccination	Europe
EF565364	Denmark	2003	Pcv2b	Pre-Vaccination	Europe
EF565350	Denmark	2003	Pcv2b	Pre-Vaccination	Europe
DQ861899	Brazil	2003	Pcv2b	Pre-Vaccination	South America
AY217743	China	2003	Pcv2b	Pre-Vaccination	Asia
DQ861897	Brazil	2003	Pcv2b	Pre-Vaccination	South America
DQ861898	Brazil	2003	Pcv2b	Pre-Vaccination	South America
AY256457	Hungary	2003	Pcv2b	Pre-Vaccination	Europe
DQ233257	Romania	2003	Pcv2b	Pre-Vaccination	Europe
AF538325	China	2003	Pcv2b	Pre-Vaccination	Asia
AY177626	China	2003	Pcv2b	Pre-Vaccination	Asia
KC835190	Brazil	2003	Pcv2b	Pre-Vaccination	South America
EF565357	Denmark	2003	Pcv2b	Pre-Vaccination	Europe
DQ915587	Greece	2003	Pcv2b	Pre-Vaccination	Europe
AY256460	Hungary	2003	Pcv2b	Pre-Vaccination	Europe
AY181945	China	2003	Pcv2b	Pre-Vaccination	Asia
AY288134	China	2003	Pcv2b	Pre-Vaccination	Asia
EU136712	Denmark	2003	Pcv2b	Pre-Vaccination	Europe
EF565345	Denmark	2003	Pcv2b	Pre-Vaccination	Europe
EU136713	Denmark	2003	Pcv2b	Pre-Vaccination	Europe
EF565347	Denmark	2003	Pcv2b	Pre-Vaccination	Europe
EF565361	Denmark	2003	Pcv2b	Pre-Vaccination	Europe
EU136716	Denmark	2003	Pcv2b	Pre-Vaccination	Europe
EF565363	Denmark	2003	Pcv2b	Pre-Vaccination	Europe
EF565346	Denmark	2003	Pcv2b	Pre-Vaccination	Europe
EF565368	Denmark	2003	Pcv2b	Pre-Vaccination	Europe
EU136715	Denmark	2003	Pcv2b	Pre-Vaccination	Europe
EU136714	Denmark	2003	Pcv2b	Pre-Vaccination	Europe
EF565348	Denmark	2003	Pcv2b	Pre-Vaccination	Europe
EF565343	Denmark	2003	Pcv2b	Pre-Vaccination	Europe
EF565353	Denmark	2003	Pcv2b	Pre-Vaccination	Europe
EF565359	Denmark	2003	Pcv2b	Pre-Vaccination	Europe
EF565352	Denmark	2003	Pcv2b	Pre-Vaccination	Europe
EF565356	Denmark	2003	Pcv2b	Pre-Vaccination	Europe
EF565367	Denmark	2003	Pcv2b	Pre-Vaccination	Europe
AY291316	China	2003	Pcv2b	Pre-Vaccination	Asia
EF565354	Denmark	2003	Pcv2b	Pre-Vaccination	Europe
EF565351	Denmark	2003	Pcv2b	Pre-Vaccination	Europe
EF565349	Denmark	2003	Pcv2b	Pre-Vaccination	Europe
EF565365	Denmark	2003	Pcv2b	Pre-Vaccination	Europe
AY391729	China	2003	Pcv2b	Pre-Vaccination	Asia
EF565358	Denmark	2003	Pcv2b	Pre-Vaccination	Europe
EF565344	Denmark	2003	Pcv2b	Pre-Vaccination	Europe
EF565342	Denmark	2003	Pcv2b	Pre-Vaccination	Europe
AY424405	Austria	2003	Pcv2b	Pre-Vaccination	Europe
HQ831524	Portugal	2003	Pcv2b	Pre-Vaccination	Europe
HQ831521	Portugal	2004	Pcv2b	Pre-Vaccination	Europe

EU346945	China	2004	Pcv2b	Pre-Vaccination	Asia
EU346945	China	2004	Pcv2b	Pre-Vaccination	Asia
AY322002	France	2004	Pcv2b	Pre-Vaccination	Europe
AY484409	Netherlands	2004	Pcv2b	Pre-Vaccination	Europe
DQ861895	Brazil	2004	Pcv2b	Pre-Vaccination	South America
DQ861896	Brazil	2004	Pcv2b	Pre-Vaccination	South America
AY536755	China	2004	Pcv2b	Pre-Vaccination	Asia
DQ861902	Brazil	2004	Pcv2b	Pre-Vaccination	South America
AY321991	France	2004	Pcv2b	Pre-Vaccination	Europe
AY484415	Netherlands	2004	Pcv2b	Pre-Vaccination	Europe
AY321990	France	2004	Pcv2b	Pre-Vaccination	Europe
KC835191	Brazil	2004	Pcv2b	Pre-Vaccination	South America
AY321997	France	2004	Pcv2b	Pre-Vaccination	Europe
AY321998	France	2004	Pcv2b	Pre-Vaccination	Europe
AY613854	China	2004	Pcv2b	Pre-Vaccination	Asia
AY321984	France	2004	Pcv2b	Pre-Vaccination	Europe
AY321985	France	2004	Pcv2b	Pre-Vaccination	Europe
AY321986	France	2004	Pcv2b	Pre-Vaccination	Europe
AY484412	Netherlands	2004	Pcv2b	Pre-Vaccination	Europe
AY321989	France	2004	Pcv2b	Pre-Vaccination	Europe
AY321988	France	2004	Pcv2b	Pre-Vaccination	Europe
AY484411	Netherlands	2004	Pcv2b	Pre-Vaccination	Europe
AY484413	Netherlands	2004	Pcv2b	Pre-Vaccination	Europe
AY484408	Netherlands	2004	Pcv2b	Pre-Vaccination	Europe
AY579893	China	2004	Pcv2b	Pre-Vaccination	Asia
AY651850	China	2004	Pcv2b	Pre-Vaccination	Asia
AY604430	China	2004	Pcv2b	Pre-Vaccination	Asia
AY321987	France	2004	Pcv2b	Pre-Vaccination	Europe
AY686764	China	2004	Pcv2b	Pre-Vaccination	Asia
AY732494	China	2004	Pcv2b	Pre-Vaccination	Asia
AY484416	Netherlands	2004	Pcv2b	Pre-Vaccination	Europe
AY321996	France	2004	Pcv2b	Pre-Vaccination	Europe
AY321995	France	2004	Pcv2b	Pre-Vaccination	Europe
AY682990	China	2004	Pcv2b	Pre-Vaccination	Asia
AY321994	France	2004	Pcv2b	Pre-Vaccination	Europe
AY321999	France	2004	Pcv2b	Pre-Vaccination	Europe
AY322001	France	2004	Pcv2b	Pre-Vaccination	Europe
AY321992	France	2004	Pcv2b	Pre-Vaccination	Europe
AY322000	France	2004	Pcv2b	Pre-Vaccination	Europe
AY641542	China	2004	Pcv2b	Pre-Vaccination	Asia
AY691679	China	2004	Pcv2b	Pre-Vaccination	Asia
AM086384	China	2004	Pcv2b	Pre-Vaccination	Asia
AY536756	China	2004	Pcv2b	Pre-Vaccination	Asia
AY682993	China	2004	Pcv2b	Pre-Vaccination	Asia
AY596823	China	2004	Pcv2b	Pre-Vaccination	Asia
AY686762	China	2004	Pcv2b	Pre-Vaccination	Asia
AY484414	Netherlands	2004	Pcv2b	Pre-Vaccination	Europe
HM038020	China	2004	Pcv2b	Pre-Vaccination	Asia
HQ831531	Portugal	2004	Pcv2b	Pre-Vaccination	Europe
HM038018	China	2005	Pcv2b	Pre-Vaccination	Asia
HM038016	China	2005	Pcv2b	Pre-Vaccination	Asia
HM038024	China	2005	Pcv2b	Pre-Vaccination	Asia
DQ141322	China	2005	Pcv2b	Pre-Vaccination	Asia

JX512857	Switzerland	2005	Pcv2b	Pre-Vaccination	Europe
AY969004	China	2005	Pcv2b	Pre-Vaccination	Asia
JX512859	Switzerland	2005	Pcv2b	Pre-Vaccination	Europe
FJ905466	SouthKorea	2005	Pcv2b	Pre-Vaccination	Asia
AY188355	China	2005	Pcv2b	Pre-Vaccination	Asia
KC835193	Brazil	2005	Pcv2b	Pre-Vaccination	South America
KC835192	Brazil	2005	Pcv2b	Pre-Vaccination	South America
FJ644561	China	2005	Pcv2b	Pre-Vaccination	Asia
HM009336	Slovakia	2005	Pcv2b	Pre-Vaccination	Europe
AY916791	China	2005	Pcv2b	Pre-Vaccination	Asia
DQ629116	USA	2005	Pcv2b	Pre-Vaccination	North America
DQ629117	USA	2005	Pcv2b	Pre-Vaccination	North America
DQ629118	USA	2005	Pcv2b	Pre-Vaccination	North America
DQ220729	Canada	2005	Pcv2b	Pre-Vaccination	North America
DQ220736	Canada	2005	Pcv2b	Pre-Vaccination	North America
DQ220731	Canada	2005	Pcv2b	Pre-Vaccination	North America
DQ220730	Canada	2005	Pcv2b	Pre-Vaccination	North America
DQ220739	Canada	2005	Pcv2b	Pre-Vaccination	North America
DQ220734	Canada	2005	Pcv2b	Pre-Vaccination	North America
DQ220728	Canada	2005	Pcv2b	Pre-Vaccination	North America
DQ220727	Canada	2005	Pcv2b	Pre-Vaccination	North America
DQ220733	Canada	2005	Pcv2b	Pre-Vaccination	North America
DQ220735	Canada	2005	Pcv2b	Pre-Vaccination	North America
DQ220732	Canada	2005	Pcv2b	Pre-Vaccination	North America
HQ713495	USA	2005	Pcv2b	Pre-Vaccination	North America
HQ148879	Cuba	2005	Pcv2b	Pre-Vaccination	South America
EF394779	Canada	2005	Pcv2b	Pre-Vaccination	North America
EF394778	Canada	2005	Pcv2b	Pre-Vaccination	North America
DQ629119	USA	2005	Pcv2b	Pre-Vaccination	North America
DQ629115	USA	2005	Pcv2b	Pre-Vaccination	North America
EF394777	Canada	2005	Pcv2b	Pre-Vaccination	North America
FJ905461	SouthKorea	2005	Pcv2b	Pre-Vaccination	Asia
FJ905462	SouthKorea	2005	Pcv2b	Pre-Vaccination	Asia
DQ220737	Canada	2005	Pcv2b	Pre-Vaccination	North America
DQ220738	Canada	2005	Pcv2b	Pre-Vaccination	North America
FJ644559	China	2005	Pcv2b	Pre-Vaccination	Asia
FJ644563	China	2005	Pcv2b	Pre-Vaccination	Asia
FJ644562	China	2005	Pcv2b	Pre-Vaccination	Asia
JX512860	Switzerland	2005	Pcv2b	Pre-Vaccination	Europe
FJ644558	China	2005	Pcv2b	Pre-Vaccination	Asia
AY849938	China	2005	Pcv2b	Pre-Vaccination	Asia
FJ644555	China	2005	Pcv2b	Pre-Vaccination	Asia
DQ104420	China	2005	Pcv2b	Pre-Vaccination	Asia
FJ905465	SouthKorea	2005	Pcv2b	Pre-Vaccination	Asia
DQ104422	China	2005	Pcv2b	Pre-Vaccination	Asia
FJ905464	SouthKorea	2005	Pcv2b	Pre-Vaccination	Asia
DQ017036	China	2005	Pcv2b	Pre-Vaccination	Asia
KF374705	Brazil	2005	Pcv2b	Pre-Vaccination	South America
HQ831529	Portugal	2005	Pcv2b	Pre-Vaccination	Europe
GU247987	China	2005	Pcv2b	Pre-Vaccination	Asia
GU247992	China	2005	Pcv2b	Pre-Vaccination	Asia
HQ831534	Portugal	2008	Pcv2b	Post-Vaccination	Europe
EU684164	Australia	2008	Pcv2b	Post-Vaccination	Oceania

EU555439	China	2008	Pcv2b	Post-Vaccination	Asia
KP231134	Italy	2008	Pcv2b	Post-Vaccination	Europe
KP231130	Italy	2008	Pcv2b	Post-Vaccination	Europe
KF742546	China	2008	Pcv2b	Post-Vaccination	Asia
FJ716703	China	2008	Pcv2b	Post-Vaccination	Asia
HM776446	China	2008	Pcv2b	Post-Vaccination	Asia
HM776445	China	2008	Pcv2b	Post-Vaccination	Asia
KP231129	Italy	2008	Pcv2b	Post-Vaccination	Europe
FJ233906	Canada	2008	Pcv2b	Post-Vaccination	North America
FJ233905	Canada	2008	Pcv2b	Post-Vaccination	North America
KP231102	Italy	2008	Pcv2b	Post-Vaccination	Europe
HQ591375	Croatia	2008	Pcv2b	Post-Vaccination	Europe
HQ591370	Croatia	2008	Pcv2b	Post-Vaccination	Europe
HQ591371	Croatia	2008	Pcv2b	Post-Vaccination	Europe
HQ591365	Croatia	2008	Pcv2b	Post-Vaccination	Europe
EU909688	Belgium	2008	Pcv2b	Post-Vaccination	Europe
HQ591377	Croatia	2008	Pcv2b	Post-Vaccination	Europe
HQ591376	Croatia	2008	Pcv2b	Post-Vaccination	Europe
FJ644930	China	2008	Pcv2b	Post-Vaccination	Asia
FJ804417	Germany	2008	Pcv2b	Post-Vaccination	Europe
GU325754	China	2008	Pcv2b	Post-Vaccination	Asia
HQ231329	Germany	2008	Pcv2b	Post-Vaccination	Europe
KP231104	Italy	2008	Pcv2b	Post-Vaccination	Europe
KP231132	Italy	2008	Pcv2b	Post-Vaccination	Europe
HQ831537	Portugal	2008	Pcv2b	Post-Vaccination	Europe
KP231105	Italy	2008	Pcv2b	Post-Vaccination	Europe
KP231133	Italy	2008	Pcv2b	Post-Vaccination	Europe
GQ174519	China	2008	Pcv2b	Post-Vaccination	Asia
KP231103	Italy	2008	Pcv2b	Post-Vaccination	Europe
KP231135	Italy	2008	Pcv2b	Post-Vaccination	Europe
FJ948167	China	2008	Pcv2b	Post-Vaccination	Asia
HM038028	China	2008	Pcv2b	Post-Vaccination	Asia
KJ128272	Lithuania	2009	Pcv2b	Post-Vaccination	Europe
HM776451	China	2009	Pcv2b	Post-Vaccination	Asia
GU083582	China	2009	Pcv2b	Post-Vaccination	Asia
GQ996404	China	2009	Pcv2b	Post-Vaccination	Asia
HM776444	China	2009	Pcv2b	Post-Vaccination	Asia
KJ128270	Lithuania	2009	Pcv2b	Post-Vaccination	Europe
KJ128273	Lithuania	2009	Pcv2b	Post-Vaccination	Europe
KP231154	Italy	2009	Pcv2b	Post-Vaccination	Europe
KP231155	Italy	2009	Pcv2b	Post-Vaccination	Europe
KJ128269	Lithuania	2009	Pcv2b	Post-Vaccination	Europe
HQ591378	Croatia	2009	Pcv2b	Post-Vaccination	Europe
JF317580	SouthKorea	2009	Pcv2b	Post-Vaccination	Asia
GU799576	USA	2009	Pcv2b	Post-Vaccination	North America
JF317576	SouthKorea	2009	Pcv2b	Post-Vaccination	Asia
JF317565	SouthKorea	2009	Pcv2b	Post-Vaccination	Asia
HQ591380	Croatia	2009	Pcv2b	Post-Vaccination	Europe
GU325766	China	2009	Pcv2b	Post-Vaccination	Asia
JF317570	SouthKorea	2009	Pcv2b	Post-Vaccination	Asia
JF317569	SouthKorea	2009	Pcv2b	Post-Vaccination	Asia
JF317572	SouthKorea	2009	Pcv2b	Post-Vaccination	Asia
JF317579	SouthKorea	2009	Pcv2b	Post-Vaccination	Asia

GQ449672	China	2009	Pcv2b	Post-Vaccination	Asia
GU325764	China	2009	Pcv2b	Post-Vaccination	Asia
JF317575	SouthKorea	2009	Pcv2b	Post-Vaccination	Asia
HQ231328	Germany	2009	Pcv2b	Post-Vaccination	Europe
HQ831538	Portugal	2009	Pcv2b	Post-Vaccination	Europe
GU938303	China	2009	Pcv2b	Post-Vaccination	Asia
HQ378159	Serbia	2009	Pcv2b	Post-Vaccination	Europe
HM003570	China	2009	Pcv2b	Post-Vaccination	Asia
HQ378160	Serbia	2009	Pcv2b	Post-Vaccination	Europe
HM003569	China	2009	Pcv2b	Post-Vaccination	Asia
KP231143	Italy	2009	Pcv2b	Post-Vaccination	Europe
HQ378161	Serbia	2009	Pcv2b	Post-Vaccination	Europe
KP231141	Italy	2009	Pcv2b	Post-Vaccination	Europe
KP231142	Italy	2009	Pcv2b	Post-Vaccination	Europe
HQ831540	Portugal	2010	Pcv2b	Post-Vaccination	Europe
JF317588	SouthKorea	2010	Pcv2b	Post-Vaccination	Asia
JF317585	SouthKorea	2010	Pcv2b	Post-Vaccination	Asia
KP231159	Italy	2010	Pcv2b	Post-Vaccination	Europe
JQ002672	China	2011	Pcv2b	Post-Vaccination	Asia
JQ181591	VietNam	2011	Pcv2b	Post-Vaccination	Asia
JQ181601	VietNam	2011	Pcv2b	Post-Vaccination	Asia
JQ181590	VietNam	2011	Pcv2b	Post-Vaccination	Asia
KJ920205	Germany	2011	Pcv2b	Post-Vaccination	Europe
KF742541	China	2011	Pcv2b	Post-Vaccination	Asia
KP231116	Italy	2011	Pcv2b	Post-Vaccination	Europe
KP231115	Italy	2012	Pcv2b	Post-Vaccination	Europe
KP231164	Italy	2012	Pcv2b	Post-Vaccination	Europe
KF732649	China	2012	Pcv2b	Post-Vaccination	Asia
KP231137	Italy	2013	Pcv2b	Post-Vaccination	Europe
JX099783	VietNam	2009	Pcv2a	Domestic	Asia
JQ181596	VietNam	2011	Pcv2a	Domestic	Asia
JQ181598	VietNam	2011	Pcv2a	Domestic	Asia
JX099784	VietNam	2010	Pcv2a	Domestic	Asia
KJ729074	India	2013	Pcv2a	Domestic	Asia
JX099780	VietNam	2009	Pcv2a	Domestic	Asia
JQ181604	VietNam	2011	Pcv2a	Domestic	Asia
JQ181606	VietNam	2011	Pcv2a	Domestic	Asia
JQ181588	VietNam	2011	Pcv2a	Domestic	Asia
JQ181602	VietNam	2011	Pcv2a	Domestic	Asia
JQ181587	VietNam	2011	Pcv2a	Domestic	Asia
HQ402903	China	2008	Pcv2a	Domestic	Asia
KJ094601	Brazil	2010	Pcv2a	Domestic	South America
KC800636	China	2012	Pcv2a	Domestic	Asia
KC800634	China	2010	Pcv2a	Domestic	Asia
JX982225	China	2011	Pcv2a	Domestic	Asia
GU001709	China	2009	Pcv2a	Domestic	Asia
JX982223	China	2011	Pcv2a	Domestic	Asia
KP231137	Italy	2013	Pcv2b	Domestic	Europe
JQ002672	China	2011	Pcv2b	Domestic	Asia
GU083582	China	2009	Pcv2b	Domestic	Asia
HM038028	China	2008	Pcv2b	Domestic	Asia
HQ378160	Serbia	2009	Pcv2b	Domestic	Europe
JQ181591	VietNam	2011	Pcv2b	Domestic	Asia

JQ181601	VietNam	2011	Pcv2b	Domestic	Asia
JQ181590	VietNam	2011	Pcv2b	Domestic	Asia
HQ831540	Portugal	2010	Pcv2b	Domestic	Europe
KF742546	China	2008	Pcv2b	Domestic	Asia
HQ591375	Croatia	2008	Pcv2b	Domestic	Europe
FJ948167	China	2008	Pcv2b	Domestic	Asia
HM003569	China	2009	Pcv2b	Domestic	Asia
KP231129	Italy	2008	Pcv2b	Domestic	Europe
KP231130	Italy	2008	Pcv2b	Domestic	Europe
KP231134	Italy	2008	Pcv2b	Domestic	Europe
KC261601	Brazil	2008	Pcv2b	Domestic	South America
HQ831538	Portugal	2009	Pcv2b	Domestic	Europe
HQ591378	Croatia	2009	Pcv2b	Domestic	Europe
HQ591370	Croatia	2008	Pcv2b	Domestic	Europe
HQ591371	Croatia	2008	Pcv2b	Domestic	Europe
HQ591365	Croatia	2008	Pcv2b	Domestic	Europe
KP231159	Italy	2010	Pcv2b	Domestic	Europe
KP231154	Italy	2009	Pcv2b	Domestic	Europe
KP231155	Italy	2009	Pcv2b	Domestic	Europe
KJ128269	Lithuania	2009	Pcv2b	Domestic	Europe
KJ128270	Lithuania	2009	Pcv2b	Domestic	Europe
JF317580	SouthKorea	2009	Pcv2b	Domestic	Asia
FJ233906	Canada	2008	Pcv2b	Domestic	North America
FJ233905	Canada	2008	Pcv2b	Domestic	North America
GU799576	USA	2009	Pcv2b	Domestic	North America
JF317576	SouthKorea	2009	Pcv2b	Domestic	Asia
JF317565	SouthKorea	2009	Pcv2b	Domestic	Asia
KJ128272	Lithuania	2009	Pcv2b	Domestic	Europe
KJ128273	Lithuania	2009	Pcv2b	Domestic	Europe
KJ920205	Germany	2011	Pcv2b	Domestic	Europe
EU909688	Belgium	2008	Pcv2b	Domestic	Europe
HQ378161	Serbia	2009	Pcv2b	Domestic	Europe
HM776444	China	2009	Pcv2b	Domestic	Asia
HQ378159	Serbia	2009	Pcv2b	Domestic	Europe
FJ716703	China	2008	Pcv2b	Domestic	Asia
HM776446	China	2008	Pcv2b	Domestic	Asia
GQ174519	China	2008	Pcv2b	Domestic	Asia
JF317588	SouthKorea	2010	Pcv2b	Domestic	Asia
HQ591380	Croatia	2009	Pcv2b	Domestic	Europe
JF317585	SouthKorea	2010	Pcv2b	Domestic	Asia
GU325766	China	2009	Pcv2b	Domestic	Asia
HM776445	China	2008	Pcv2b	Domestic	Asia
KF732649	China	2012	Pcv2b	Domestic	Asia
EU555439	China	2008	Pcv2b	Domestic	Asia
HM003570	China	2009	Pcv2b	Domestic	Asia
JF317570	SouthKorea	2009	Pcv2b	Domestic	Asia
JF317569	SouthKorea	2009	Pcv2b	Domestic	Asia
JF317572	SouthKorea	2009	Pcv2b	Domestic	Asia
HM776451	China	2009	Pcv2b	Domestic	Asia
KP231105	Italy	2008	Pcv2b	Domestic	Europe
KP231104	Italy	2008	Pcv2b	Domestic	Europe
KP231103	Italy	2008	Pcv2b	Domestic	Europe
KP231102	Italy	2008	Pcv2b	Domestic	Europe

Supplementary table 1

KP231135	Italy	2008	Pcv2b	Domestic	Europe
KP231132	Italy	2008	Pcv2b	Domestic	Europe
KP231133	Italy	2008	Pcv2b	Domestic	Europe
HQ831534	Portugal	2008	Pcv2b	Domestic	Europe
HQ591377	Croatia	2008	Pcv2b	Domestic	Europe
HQ591376	Croatia	2008	Pcv2b	Domestic	Europe
FJ644930	China	2008	Pcv2b	Domestic	Asia
KP231141	Italy	2009	Pcv2b	Domestic	Europe
KP231143	Italy	2009	Pcv2b	Domestic	Europe
KP231142	Italy	2009	Pcv2b	Domestic	Europe
HQ831537	Portugal	2008	Pcv2b	Domestic	Europe
HQ231329	Germany	2008	Pcv2b	Domestic	Europe
FJ804417	Germany	2009	Pcv2b	Domestic	Europe
JF317579	SouthKorea	2009	Pcv2b	Domestic	Asia
KF742541	China	2011	Pcv2b	Domestic	Asia
KP231164	Italy	2012	Pcv2b	Domestic	Europe
KP231115	Italy	2012	Pcv2b	Domestic	Europe
GQ449672	China	2009	Pcv2b	Domestic	Asia
GU938303	China	2009	Pcv2b	Domestic	Asia
HQ231328	Germany	2009	Pcv2b	Domestic	Europe
GQ996404	China	2009	Pcv2b	Domestic	Asia
GU325754	China	2008	Pcv2b	Domestic	Asia
1397-2011	Italy	2011	Pcv2b	Domestic	Europe
GU325764	China	2009	Pcv2b	Domestic	Asia
JF317575	SouthKorea	2009	Pcv2b	Domestic	Asia
EU684164	Australia	2008	Pcv2b	Domestic	Oceania
JF827599	China	2010	Pcv2d	Domestic	Asia
JX982220	China	2011	Pcv2d	Domestic	Asia
KJ680355	China	2010	Pcv2d	Domestic	Asia
KC823057	China	2012	Pcv2d	Domestic	Asia
KP231168	Italy	2014	Pcv2d	Domestic	Europe
JX682407	China	2011	Pcv2d	Domestic	Asia
JX204386	China	2012	Pcv2d	Domestic	Asia
GQ359010	China	2008	Pcv2d	Domestic	Asia
KJ680343	China	2010	Pcv2d	Domestic	Asia
HQ693093	China	2010	Pcv2d	Domestic	Asia
JF272498	China	2009	Pcv2d	Domestic	Asia
JX948785	China	2011	Pcv2d	Domestic	Asia
GU325760	China	2009	Pcv2d	Domestic	Asia
KC821783	China	2013	Pcv2d	Domestic	Asia
JX948776	China	2011	Pcv2d	Domestic	Asia
KM245558	China	2014	Pcv2d	Domestic	Asia
JX535296	USA	2012	Pcv2d	Domestic	North America
HM776441	China	2009	Pcv2d	Domestic	Asia
FJ870973	China	2008	Pcv2d	Domestic	Asia
FJ870971	China	2008	Pcv2d	Domestic	Asia
KF742551	China	2012	Pcv2d	Domestic	Asia
FJ948168	China	2008	Pcv2d	Domestic	Asia
KC753772	China	2012	Pcv2d	Domestic	Asia
KC800646	China	2011	Pcv2d	Domestic	Asia
KT216676	Brazil	2012	PCV2b	wildboar	South America
JN006452	Romania	2008	PCV2b	wildboar	Europe
JN006454	Romania	2008	PCV2b	wildboar	Europe

JN006458	Romania	2008	PCV2b	wildboar	Europe
JN382167	Romania	2011	PCV2b	wildboar	Europe
JN006456	Romania	2008	PCV2b	wildboar	Europe
JN006451	Romania	2008	PCV2b	wildboar	Europe
JN006460	Romania	2010	PCV2b	wildboar	Europe
KP768483	Slovakia	2012	PCV2b	wildboar	Europe
KC620508	SouthKorea	2012	PCV2d	wildboar	Asia
KP768484	Slovakia	2012	PCV2b	wildboar	Europe
JN382183	Romania	2011	PCV2d	wildboar	Europe
KC620518	SouthKorea	2012	PCV2b	wildboar	Asia
JN382159	Romania	2011	PCV2b	wildboar	Europe
JN006457	Romania	2008	PCV2b	wildboar	Europe
KP768486	Slovakia	2012	PCV2b	wildboar	Europe
JN382169	Romania	2011	PCV2b	wildboar	Europe
JN006465	Romania	2010	PCV2a	wildboar	Europe
JN382172	Romania	2011	PCV2b	wildboar	Europe
JN382182	Romania	2011	PCV2b	wildboar	Europe
KT216674	Brazil	2012	PCV2b	wildboar	South America
JN382158	Romania	2011	PCV2d	wildboar	Europe
JN382161	Romania	2011	PCV2a	wildboar	Europe
JN006447	Romania	2009	PCV2d	wildboar	Europe
HQ591381	Croatia	2009	PCV2a	wildboar	Europe
KC620520	SouthKorea	2011	PCV2b	wildboar	Asia
KT216672	Brazil	2012	PCV2b	wildboar	South America
KP768478	Slovakia	2012	PCV2b	wildboar	Europe
JN006455	Romania	2008	PCV2b	wildboar	Europe
JN382181	Romania	2011	PCV2b	wildboar	Europe
KC620505	SouthKorea	2012	PCV2b	wildboar	Asia
KC620507	SouthKorea	2011	PCV2b	wildboar	Asia
JN382173	Romania	2011	PCV2b	wildboar	Europe
KP768476	Slovakia	2012	PCV2b	wildboar	Europe
JN006444	Romania	2009	PCV2d	wildboar	Europe
JN382174	Romania	2011	PCV2b	wildboar	Europe
KP768470	Slovakia	2012	PCV2b	wildboar	Europe
KC620503	SouthKorea	2011	PCV2b	wildboar	Asia
JN382165	Romania	2011	PCV2b	wildboar	Europe
JN382176	Romania	2011	PCV2b	wildboar	Europe
KT216677	Brazil	2012	PCV2b	wildboar	South America
JN382184	Romania	2011	PCV2b	wildboar	Europe
JN382177	Romania	2011	PCV2a	wildboar	Europe
KC620510	SouthKorea	2011	PCV2d	wildboar	Asia
KC620511	SouthKorea	2010	PCV2b	wildboar	Asia
KC261600	Brazil	2008	PCV2b	wildboar	South America
JN382180	Romania	2011	PCV2b	wildboar	Europe
JN382178	Romania	2011	PCV2a	wildboar	Europe
JN382170	Romania	2011	PCV2d	wildboar	Europe
KP768467	Slovakia	2012	PCV2a	wildboar	Europe
KP768477	Slovakia	2012	PCV2b	wildboar	Europe
KT216673	Brazil	2012	PCV2b	wildboar	South America
KP768480	Slovakia	2012	PCV2a	wildboar	Europe
KC620512	SouthKorea	2012	PCV2b	wildboar	Asia
KP768468	Slovakia	2012	PCV2b	wildboar	Europe
JN006448	Romania	2010	PCV2d	wildboar	Europe

JN382175	Romania	2011	PCV2b	wildboar	Europe
KC620504	SouthKorea	2011	PCV2d	wildboar	Asia
KP768472	Slovakia	2012	PCV2b	wildboar	Europe
JN382179	Romania	2011	PCV2d	wildboar	Europe
KP768471	Slovakia	2012	PCV2b	wildboar	Europe
KC620517	SouthKorea	2011	PCV2b	wildboar	Asia
JN006449	Romania	2008	PCV2d	wildboar	Europe
JN006445	Romania	2010	PCV2d	wildboar	Europe
KP768482	Slovakia	2012	PCV2b	wildboar	Europe
KC620516	SouthKorea	2011	PCV2b	wildboar	Asia
KP768475	Slovakia	2012	PCV2a	wildboar	Europe
JN382162	Romania	2011	PCV2a	wildboar	Europe
JN006462	Romania	2010	PCV2b	wildboar	Europe
KC620509	SouthKorea	2010	PCV2b	wildboar	Asia
JN006450	Romania	2010	PCV2d	wildboar	Europe
JN006461	Romania	2010	PCV2b	wildboar	Europe
JN382157	Romania	2011	PCV2a	wildboar	Europe
KC620502	SouthKorea	2011	PCV2b	wildboar	Asia
JN382164	Romania	2011	PCV2b	wildboar	Europe
JN006463	Romania	2010	PCV2b	wildboar	Europe
JN382163	Romania	2011	PCV2d	wildboar	Europe
KC620513	SouthKorea	2012	PCV2b	wildboar	Asia
KT216675	Brazil	2012	PCV2b	wildboar	South America
KP768473	Slovakia	2012	PCV2b	wildboar	Europe
JN382168	Romania	2011	PCV2b	wildboar	Europe
KP768481	Slovakia	2012	PCV2b	wildboar	Europe
KC261601	Brazil	2008	PCV2b	wildboar	South America
KP768469	Slovakia	2012	PCV2b	wildboar	Europe
KP768479	Slovakia	2012	PCV2b	wildboar	Europe
KP768474	Slovakia	2012	PCV2a	wildboar	Europe
KC620519	SouthKorea	2011	PCV2b	wildboar	Asia
JN382171	Romania	2011	PCV2b	wildboar	Europe
JN382160	Romania	2011	PCV2a	wildboar	Europe
JN006443	Romania	2008	PCV2d	wildboar	Europe
JN006464	Romania	2010	PCV2b	wildboar	Europe
KC620506	SouthKorea	2011	PCV2b	wildboar	Asia
JN006453	Romania	2008	PCV2b	wildboar	Europe
JN006446	Romania	2010	PCV2d	wildboar	Europe
KC620515	SouthKorea	2012	PCV2b	wildboar	Asia
KP768485	Slovakia	2012	PCV2b	wildboar	Europe
KC620514	SouthKorea	2010	PCV2b	wildboar	Asia
JN382166	Romania	2015	PCV2b	wildboar	Europe
JN006459	Romania	2010	PCV2b	wildboar	Europe
KR559725	Italy	2011	PCV2b	wildboar	Europe
KR559724	Italy	2011	PCV2d	wildboar	Europe
KR559723	Italy	2011	PCV2b	wildboar	Europe
KR559722	Italy	2011	PCV2b	wildboar	Europe
KR559721	Italy	2011	PCV2b	wildboar	Europe
KR559720	Italy	2011	PCV2b	wildboar	Europe
KR559719	Italy	2011	PCV2b	wildboar	Europe
KR559718	Italy	2011	PCV2b	wildboar	Europe
KR559717	Italy	2011	PCV2b	wildboar	Europe
KR559716	Italy	2011	PCV2b	wildboar	Europe

KR559715	Italy	2011	PCV2b	wildboar	Europe
KR559714	Italy	2011	PCV2b	wildboar	Europe
KR559713	Italy	2011	PCV2b	wildboar	Europe
KR559712	Italy	2011	PCV2d	wildboar	Europe
KR559711	Italy	2011	PCV2d	wildboar	Europe
KR559710	Italy	2011	PCV2b	wildboar	Europe
KR559709	Italy	2011	PCV2d	wildboar	Europe
KR559708	Italy	2011	PCV2b	wildboar	Europe
KR559707	Italy	2011	PCV2b	wildboar	Europe
KR559706	Italy	2012	PCV2b	wildboar	Europe
KR559705	Italy	2010	PCV2b	wildboar	Europe
KR559704	Italy	2010	PCV2b	wildboar	Europe
KR559703	Italy	2010	PCV2b	wildboar	Europe
KR559702	Italy	2010	PCV2b	wildboar	Europe
KR559701	Italy	2011	PCV2b	wildboar	Europe
KR559700	Italy	2009	PCV2b	wildboar	Europe
KR559699	Italy	2009	PCV2b	wildboar	Europe
KR559698	Italy	2009	PCV2b	wildboar	Europe
