# Supplementary material for: Porcine circovirus type 2 (PCV2) evolution before and after the vaccination introduction: A large scale epidemiological study
Source: Sci Rep. 2016 Dec 19;6:39458. doi: 10.1038/srep39458 (PMC5171922; doi:10.1038/srep39458)
Supplement: Supplementary Table 2 [file srep39458-s4.pdf]

Porcine circovirus type 2 (PCV2) evolution before and after the vaccination introduction. A large scale epidemiological study.

Giovanni Franzo[*#1], Claudia Maria Tucciarone[#1], Mattia Cecchinato[1] and Michele Drigo[1].

[1]University of Padua, Legnaro (PD), Italy;

Supplementary table 2.

	Pcv2a		Total
**Area**	Pre-Vaccination	Post-Vaccination	
**Africa**	1		**1**
**Asia**	33	48	**81**
**Europe**	26	14	**40**
**North America**	46	78	**124**
**Oceania**	7		**7**
**South America**		5	**5**
**Total**	**113**	**145**	**258**

	Pcv2b		Total
**Area**	Pre-Vaccination	Post-Vaccination	
**Asia**	50	36	**86**
**Europe**	77	44	**121**
**North America**	22	3	**25**
**Oceania**		1	**1**
**South America**	14		**14**
**Total**	**163**	**84**	**247**

	PCV2a-PCV2b-PCV2d		
**Area**	Wildboar	Domestic	**Total**
**Asia**	19	75	**94**
**Europe**	100	45	**145**
**North America**		4	**4**
**Oceania**		1	**1**
**South America**	8	2	**10**
**Total**	**127**	**127**	**254**

Supplementary table 2. Count of strain geographic origin grouped in database 1-2 (i.e. PCV2a before and after vaccination introduction) (a), 3-4 (i.e. PCV2b before and after vaccination introduction) (b) and 5-6 (PCV2 strains detected in domestic pigs and wild boars)
